# On the physical mechanisms underlying single molecule dynamics in simple liquids

**DOI:** 10.1038/s41598-021-82112-8

**Published:** 2021-01-28

**Authors:** Russell G. Keanini, Jerry Dahlberg, Peter T. Tkacik

**Affiliations:** Department of Mechanical Engineering, University of North Carolina at Charlotte, Charlotte, 28078 USA

**Keywords:** Engineering, Physics

## Abstract

Physical arguments and comparisons with published experimental data suggest that in simple liquids: (i) single-molecule-scale viscous forces are produced by temperature-dependent London dispersion forces, (ii) viscosity decay with increasing temperature reflects electron cloud compression and attendant suppression of electron screening, produced by increased nuclear agitation, and (iii) temperature-dependent self-diffusion is driven by a narrow band of phonon frequencies lying at the low-frequency end of the solid-state-like phonon spectrum. The results suggest that collision-induced electron cloud distortion plays a decisive role in single molecule dynamics: (i) electron cloud compression produces short-lived repulsive states and single molecule, self-diffusive hops, while (ii) shear-induced distortion generates viscosity and single-molecule-scale viscous drag. The results provide new insight into nonequilibrium molecular dynamics in nonpolar, nonmetallic liquids.

## Introduction

Gaining a deeper understanding of single molecule dynamics in liquids bears on fundamental problems in chemical reaction kinetics^1,2^, sub-cellular water transport^3,4^ and biomass transfer^5,6^, detection of cosmic particles and radiation^7^, dark matter detection^8–10^, detection of collision products in high energy physics^7^, corrosion^11^, and weathering of terrestrial and extra-terrestrial surface rock^12^. The problem has attracted the attention of luminaries like Einstein^13,14^, Perrin^15^, Laundau^16^, Prigogine^17^, and Feynman^18^. Nevertheless, the physical mechanisms that determine single molecule motion in liquids remain poorly understood.

A variety of experimental and theoretical approaches have been developed for studying molecular dynamics in liquids. Experimental techniques include light and particle scattering^19–23^ which probes dynamic responses over single- to multiple-molecule length scales, and sub-collision and longer $\left( t \ge \mathrm {O} \left( 10^{-14} \ \mathrm {s} \right) \right)$ time scales. Photonic techniques^24–28^ are capable of exposing intramolecular dynamics on femtosecond time scales $\left( \mathrm {t} = \mathrm {O} \left( 10^{-15} \ \mathrm {s} \right) \right) .$ Molecular dynamics simulations provide a computational approach for probing each of these scales^29–31^.

Theoretical modeling drives and allows interpretation of typically complicated experimental observations. Over multi-molecule length scales and multiple-collision time scales, molecular hydrodynamics^20–22,31–34^ successfully connects observed spectral responses of dense fluids to the continuum Navier-Stokes (NS) equations. However, on single-molecule length scales and collision- and sub-collision time scales, mapping molecular-scale response into generalized NS models requires time- and space-dependent transport coefficients^20,22,33–35^, revealing our poor understanding of single-molecule liquid-state dynamics.

For atomic, and small polyatomic, nominally spherical, nonpolar liquids, Langevin (LE) models^22,33,34^ provide a powerful, particle-based framework for tackling molecular dynamics problems, both under classical conditions—where the dynamical processes of interest take place on time scales exceeding the ’dispersion time scale’, $\tau _d = O \left( 10^{-16} \ \mathrm {s} \right) ,$ see below—and under conditions where quantum smearing of the dynamics becomes important^33,36^. Generally speaking, LE models are suitable for particle dynamics problems characterized by short-time scale random forcing and longer-time scale non-random dynamical dissipation, as well as by possible external forcing.

This paper presents three results, which together, provide new insight into the dynamics of single molecules in nonpolar, nonmetallic liquids:

(a) A simple physical model is proposed which suggests that: (i) liquid-state viscosity is produced by temperature-dependent London dispersion forces, and (ii) viscosity decay with increasing temperature reflects decreased electron screening of nuclear charge. Comparison of predicted and experimentally observed viscosities^22,37^ for liquid Ne, Ar, Kr, Xe, $\mathrm {N_2},$ $\mathrm {O_2} ,$ and $\mathrm {CH_4} ,$ support the proposed physical picture.

(b) A Langevin model of sub-collision time scale, single molecule dynamics, which explicitly accounts for solid-state-like phonon modes, leads to a physically consistent explanation for self- diffusion coefficients measured in liquid Ar, Kr, and Xe^38^. The model suggests that on time scales ranging from the Frenkel scale, $\tau _F = O \left( 10^{-14} \ \mathrm {s} \right) ,$ down to the fast dispersion scale, $\tau _d = O \left( 10^{-16} \ \mathrm {s} \right) ,$ molecular dynamics in simple liquids is solid-like, and thus dominated by phonon modes, consistent with the equilibrium statistical mechanics picture presented by^39,40^. In addition, the model indicates that the random diffusional hopping of individual molecules is produced by a narrow band of phonon modes lying near the low-frequency end of the phonon spectrum, $\omega _c \sim \omega _F = 2 \pi / \tau _F .$

[Note, for context, $\tau _F$ is approximately an order of magnitude shorter than the characteristic intermolecular collision time scale, $\tau _c = O \left( 10^{-13} \ \mathrm {s} \right) .$ In order to provide a physical feel for the important time scales in this problem, we will often use those associated with liquid Ar. In addition, the term ’molecular’ will refer to monatomic as well as small, polyatomic liquids.]

(c) A set of time scale-dependent Langevin equations are proposed for describing single molecule dynamics in simple, non-polar liquids. The equations apply over the poorly characterized sub-collision time scale, $\tau _d \lesssim t \lesssim \tau _c ,$ incorporate the above results, and represent best-guess extrapolations of well-established dynamics on longer time scales.

As a consequence of the modeling, experimental comparisons, and consistency checks that are presented, we arrive at a preliminary picture of the decisive role apparently played by collision-induced electron cloud distortion in single molecule dynamics. Specifically, arguments and evidence are presented suggesting that phonon-induced electron cloud compression forces colliding molecular pairs into short-lived repulsive states, producing, in turn, single molecule, self-diffusive hops. In addition, we propose that nonequilibrium, shear-induced, ’tangential’ electron cloud distortion generates viscosity and single molecule scale, resistive viscous forces. In order to support the hypothesis that viscosity arises on single molecule scales (SMS), the paper first derives SMS Navier–Stokes equations, describing the ensemble average, *field-based* dynamics of (Newtonian) SMS fluid systems. Given these, a scaling estimate of SMS viscosity, based on the Green-Kubo viscosity relation^35^, then leads to a parametrically correct solution for SMS viscosity, obtained in terms of the SMS shear stress (i.e., the ensemble average, SMS transverse current correlation^22,34^).

## Results, methods and discussion

### Dispersion forces and electron screening determine temperature-dependent dynamic viscosity

As a measurable property determined by molecular-scale processes, viscosity provides a window into molecular dynamics. Here, we study temperature-dependent viscosities observed in liquid Ne, Ar, Kr, Xe, $\mathrm {N_2} ,$ $\mathrm {O_2} ,$ and $\mathrm {CH_4 },$ at fixed pressures, over the temperature ranges on which each specie exists as a liquid^22,37^.

The corresponding states principle^22,41,42^ provides the basis for our argument. The simplest form of the principle postulates that viscosity is determined by a characteristic intermolecular potential energy, $\epsilon ,$ a characteristic intermolecular length scale, $\sigma ,$ the molecular mass, *M*,  and a specie-dependent temperature-scale, $\epsilon / k_B :$

1 $$\begin{aligned} \mu = f \left( T , M \epsilon , \sigma , k_B \right) \end{aligned}$$

where, on dimensional grounds, *M* can be grouped with $\epsilon .$ Dimensional analysis allows restatement of (1) in nondimensional form:

2 $$\begin{aligned} {\tilde{\mu }} = {\tilde{f}} \left( {\tilde{T}} \right) \end{aligned}$$

where ${\tilde{\mu }} = \mu / \sqrt{M \epsilon } / \sigma ^{2} ,$ ${\tilde{T}} = T / \left( \epsilon / k_B \right) ,$ $k_B$ is Boltzmann’s constant, and ${\tilde{f}}$ represents the experimentally determined correlation. The principle holds nominally well^22,41^ in nonpolar atomic and diatomic liquids that are well-modeled by the Lennard–Jones potential^22,41^. More comprehensive correlations incorporating quantum (low temperature and small mass) effects and information on the shape of the intermolecular potential have been proposed^42^.

In order to derive what turns out to be a simple physical model for predicting viscosity in nonpolar liquids, we proceed in three steps. First, a scaling argument is used to place the corresponding states principle on a physical basis, leading to an approximate relationship for $\mu :$

3 $$\begin{aligned} \mu \approx \frac{\sqrt{ \epsilon M}}{\sigma ^2} \end{aligned}$$

Focusing on nonpolar liquids subject to London dispersion forces, we then follow^41^ and state the intermolecular energy, $$ \epsilon ,$$ in terms of specie polarizability, $$ \alpha ,$$ and the separation, $$ r_{ab} , $$ between colliding molecular pairs. Finally, collision-induced, and temperature-dependent polarization is stated in terms a mean, temperature-dependent electron cloud distortion, $$ \delta \sigma \left( T \right) . $$ Importantly, in order to obtain a viscosity model consistent with available measurements^22^, we propose that electron cloud distortion, $$ \delta \sigma \left( T \right) ,$$ decreases linearly with temperature.

Confine attention to classical conditions, assume pair-wise intermolecular collisions—see Note (i) in the final section below, neglect non-spherical shape effects^42^ on the (pair-wise) intermolecular potential, and focus on simple, nonmetallic liquids, i.e., those composed of nonpolar molecules having nominally spherical, localized electron distributions^43^. Under these conditions, the *attractive* potential between colliding molecular pairs is wholly determined by London dispersion^41,44^.

On time scales longer than the dispersion time scale, $\tau _d = \mathrm {O} \left( 10^{-16} \ \mathrm {s} \right)$ - the scale on which electron distributions oscillate^45^ - but shorter than the intermolecular collision time scale, $\tau _c = \mathrm {O} \left( 10^{-13} \ \mathrm {s} \right) ,$ two-body interactions dominate three- and higher-order interactions; again, see Note i) in the final section. Due to high-frequency phonon modes, $\omega > 2 \pi \tau _F^{-1} = \mathrm {O} \left( 10^{14} \ \mathrm {s^{-1}} \right) ,$ we assume that viscosity emerges on an intermediate time scale, $\tau _v ,$ where $\tau _d<< \tau _v<< \tau _c .$ As shown by^46^, a modified Stokes-Einstein relation,

4 $$\begin{aligned} D = \frac{2 k_B T}{ n' \pi \mu \sigma f' } \end{aligned}$$

connects diffusion of small and medium sized molecules (in water and carbon tetrachloride^46^) to the viscosity of the solvent liquids. Here, *D* is the diffusion coefficient, $\mu$ is the dynamic viscosity, $f'$ is a molecular-shape-dependent factor, and $n'$ is a correction factor, ranging from approximately 2 to 6, and accounting for a mix of slip- and no-slip flow conditions on a molecule’s surface.

Importantly, (4) implies that single molecule dynamics can be modeled using the simple, memory free Langevin equation:

5 $$\begin{aligned} M \frac{ d {\mathbf {v}} \left( t \right) }{dt} = - 3 \pi \sigma _m \mu {\mathbf {v}} + \mathbf {F_R} \left( t \right) \end{aligned}$$

where $\sigma _m = n'f' \sigma / 6$ is the effective molecular diameter; see, e.g.,^47^. Here, ${\mathbf {v}} \left( t \right)$ is the instantaneous velocity of the molecule and $\mathbf {F_R} \left( t \right)$ is the instantaneous random force on the molecule. Thus, on time scales of order $\tau _v = \mathrm {O} \left( 10^{-15} \ \mathrm {s} \right) ,$ and longer, we argue that the rate of work done on an individual molecule by the dispersion force is dissipated by viscous dissipation:

6 $$\begin{aligned} \frac{\epsilon }{\sigma } \cdot \delta r_{nuc} \cdot \tau _v^{-1} \approx \mu \frac{u_{nuc}}{\sigma } \cdot \sigma ^2 \cdot \delta r_{nuc} \cdot \tau _v^{-1} \end{aligned}$$

where spatial derivatives are approximated as $1 / \sigma ,$ the characteristic speed of the nucleus is given by $u_{nuc} \approx \sqrt{\epsilon / M },$ the characteristic nuclear displacement over $\tau _v$ is represented as $\delta r_{nuc} ,$ and the nominal surface area of the molecule is on the order of $\sigma ^2 .$ Solving (6) for the viscosity then leads to (3).

### Dispersion forces determine viscosity and increased nuclear agitation with temperature compress electron clouds, suppressing viscosity

In simple liquids subject to London interactions, the energy scale, $\epsilon ,$ is approximately determined by^41,44^

7 $$\begin{aligned} \epsilon _d = \frac{3}{4} h \nu _o \frac{\alpha ^2}{r_{ab}^6} \end{aligned}$$

where $h \nu _o$ is the ground state energy of an isotropic quantum oscillator, $\alpha$ is the polarizability, and $r_{ab}$ is again the separation between the molecular pair’s nuclei. This expression follows from assuming that pair-wise molecular collisions correspond to weak interactions between isotropic quantum oscillators^41,44^. As an initial consistency check, Supplement 4 compares estimated and experimental kinematic viscosities, $\nu = \mu / \rho ,$ for a set of simple liquids, where $\nu$ estimates use London’s rigorous second order quantum perturbation model^41,44^, $\epsilon = C / r_{{\mathscr {A}} {\mathscr {B}}}^6$ in (3), and where *C* is the attractive constant.

Since (7) allows intuitive derivation and interpretation of the viscosity estimate presented here, as well as exposing the apparent central role of electron cloud distortion in viscosity generation, we use (7) to estimate $\epsilon .$ Polarizability is given approximately by^41^

8 $$\begin{aligned} \alpha = \frac{4}{9 a_o } \sum _{i=1}^n \left( \overline{r_i^2} \right) ^2 \end{aligned}$$

where the sum is taken over the principle quantum energy levels of a given molecule, $$ \overline{r_i^2} $$ is the average squared displacement of the electrons in $$ i^{th} $$ shell (induced by an external electric field), and $$a_o $$ is the Bohr radius. In detail, $$ \overline{r_i^2}, $$ follows from introduction of Slater orbitals^41,48^:

9 $$\begin{aligned} \overline{r_i^2}=\left[ \frac{n_i^* }{ 2\left( Z - S_i \right) } \right] ^2 \left( 2 n_i^* + 1 \right) \left( 2 n_i^* + 2 \right) a_o^2 \end{aligned}$$

where $Z - S_i$ is the effective nuclear charge of the $i^{th}$ shell, *Z* is the nuclear charge, $S_i$ is the associated screening constant, and $n_i^*$ the effective principle quantum number.

Focusing on Ne, Ar, Kr, Xe, $\mathrm {O_2} ,$ and $\mathrm {N_2},$ we label the sum of mean squared electron displacements as

10 $$\begin{aligned} \frac{1}{n} \sum _{i=1}^n \left( \overline{r_i^2} \right) ^2 = \delta \sigma ^4 \left( T \right) \end{aligned}$$

where

11 $$\begin{aligned} \delta \sigma \left( T \right) = \left[ n^{-1} \sum _{i=1}^n \left( \overline{r_i^2} \right) ^2 \right] ^{1/4} \end{aligned}$$

represents the average collision-induced distortion of all the electrons in a molecule, and where we assume that $\delta \sigma \left( T \right)$ is temperature-dependent.

Over the narrow temperature ranges on which each of these species exist as a liquid, and based on the observation that liquid viscosities decrease with increasing temperature^22^, we introduce an ansatz that the mean electron distortion decreases linearly with temperature:

12 $$\begin{aligned} \delta \sigma \left( T \right) = \delta \sigma _v \left( 1 - \epsilon _T \right) = \delta \sigma _v \left( 1 - \frac{T_v-T}{T_v} \right) \end{aligned}$$

where $\delta \sigma _v = \delta \sigma \left( T_v \right) ,$ is the mean displacement at characteristic temperature, $T_v = \epsilon / k_B ,$ and $\epsilon _T = \left( T_v - T \right) / T_v .$

Physically, and in light of (9) and the results below, this guess suggests that electron screening *decreases* with increasing temperature, consistent with behavior observed in deuterated metals^49^. Since kinetic energy of both nuclei and electrons increase with rising temperature, where the latter presumably enhances screening, the suppression of screening apparently reflects increased nuclear agitation; intensifying agitation, under spatially packed conditions effectively thins surrounding electron clouds. A similar mechanism has recently been proposed in metallic glasses^50^, and may underlie atomization of vapor phase molecular clusters as $T \rightarrow T_v = \epsilon / k_B ,$ where atomization reflects nuclear kinetic energy overtaking intermolecular dispersion forces.

Next, approximate $\delta \sigma \left( T \right) = \delta \sigma _v \left( 1 - \epsilon _T \right)$ as $\delta \sigma \left( T \right) = \delta \sigma _v \exp \left( - \epsilon _T \right) = \delta \sigma _v e^{1} \exp \left( -T^* \right) ,$ where $T^* = T / T_v = T / \left( \epsilon / k_B \right) ,$ is the dimensionless temperature defined in the corresponding states correlation. Since the maximum magnitude of $\epsilon _T$ is on the order of 0.3 for the set of liquids considered, save oxygen, the maximum error introduced by replacing $\left( 1 - \epsilon _T \right)$ with $\exp \left( - \epsilon _T \right)$ is on the order of 10 %.

Using $\epsilon _d$ in (7) for $\epsilon$ in (3), the definition in (11) for the mean electron distortion, and the exponential approximation above for the assumed linear temperature variation in $\delta \sigma \left( T \right) ,$ leads to an approximate expression for the temperature-dependent viscosity for simple liquids:

13 $$\begin{aligned} \mu \left( T^* \right) \approx C_o \exp \left( -4 T^* \right) \end{aligned}$$

where $C_o = \sqrt{ 243 h \nu _o e^8 \left( \delta \sigma _v / \sigma _o \right) ^8 M /16 } / \sigma _o^2 ,$ and where $a_o$ is approximated as $\sigma _o /2 ,$ and $\sigma _o$ corresponds, e.g., to the molecular diameter at the specie melting point.

### Comparison of theoretical and observed viscosities

In order to allow comparison of theoretical, temperature-dependent viscosities, as given by (13), with experimentally measured viscosities, we define the dimensionless viscosity for liquid $\kappa ,$ $\mu _{\kappa }^* \left( T^* \right) = \mu _{\kappa } \left( T^* \right) / \mu _{m, \kappa } ,$ yielding

14 $$\begin{aligned} \mu _{\alpha }^* \left( T^* \right) = c_{\kappa }^* \exp \left( -4 T^* \right) \end{aligned}$$

where $c_{\kappa }^* = \mu _{\kappa }^* \left( T_{min, \kappa }^* \right) \exp \left( 4 T_{min, \kappa }^* \right) ,$ and where $\mu _{\kappa }^* \left( T_{min, \kappa }^* \right)$ is the measured dimensionless viscosity for fluid $\kappa$ at the minimum dimensionless temperature, $T_{min, \kappa }^*$ at which $\mu _{\kappa }^*$ is measured. Note, due to the approximations used to obtain the constant $C_o$ above, plotted viscosity estimates obtained using (13) exhibit the appropriate decay with temperature, but are displaced by a (nominally) fixed magnitude from measured viscosities.

**Figure 1:**
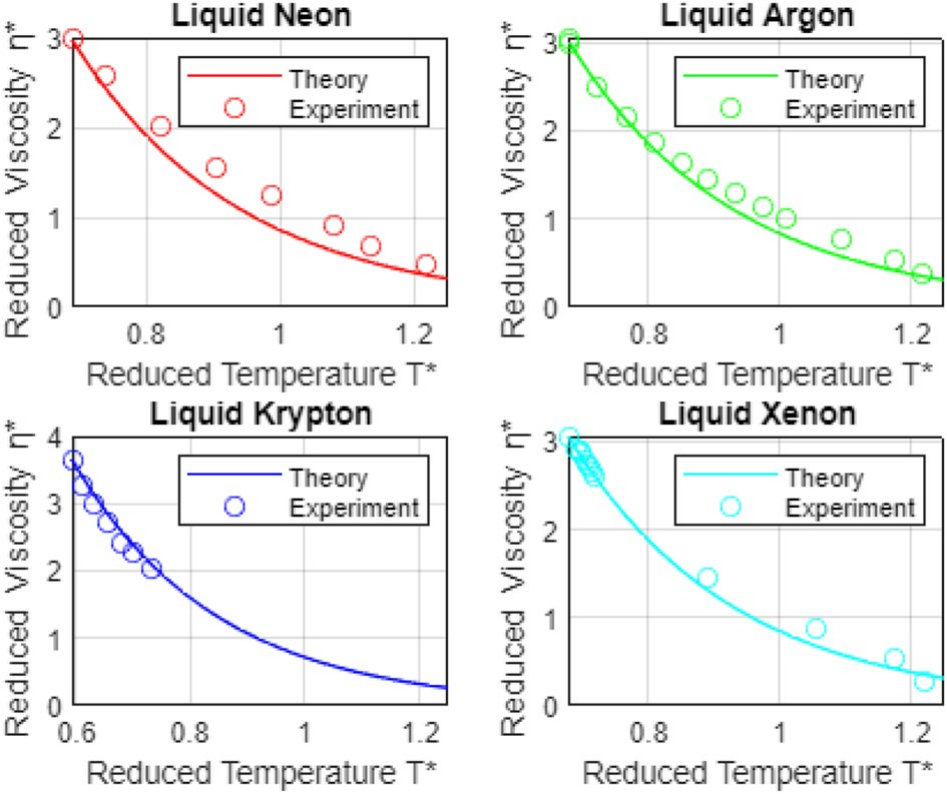
Temperature-dependent viscosity for noble liquids. The proposed model assumes: (i) dominant pairwise, dispersive, intermolecular interactions—see the scaling argument (i) in the final section, and (ii) that the average collision-induced distortion of the molecule’s electrons, $\delta \sigma \left( T \right) = \left[ \sum _{i=1}^n \left( \overline{r_i^2} \right) ^2 \right] ^{1/4},$ decays linearly with temperature. The second assumption suggests that electron screening *decreases* with increasing temperature—consistent with^49,50^—and, in turn, that thermally-driven nuclear motion dominates presumed enhanced electron shrouding of the nucleus. For an explanation of experimental conditions and definitions of dimensionless variables, see the caption to Fig. 2. Sources for the experimental viscosity data shown are as follows: Ne^51^; Ar^52^; Kr^53^; Xe^54^. For a discussion of estimated experimental uncertainties, please see the text.

**Figure 2:**
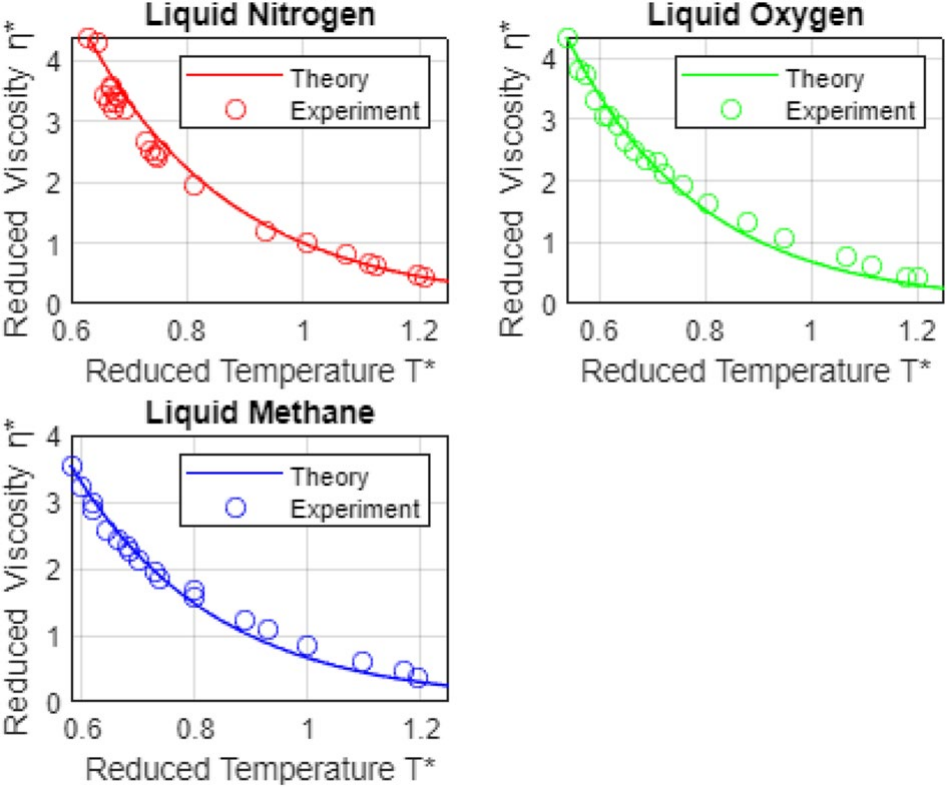
Temperature-dependent liquid viscosities for liquid $\mathrm {N_2} ,$ $\mathrm {O_2} ,$ and $\mathrm {CH_4} .$ *Notes* (a) Experimental viscosity for specie $\kappa$ is nondimensionalized using the viscosity scale $\mu _{scale, \kappa } = \sqrt{M_{\kappa } \epsilon _{min, \kappa } } \sigma _{min, \kappa }^2 ,$ where $\epsilon _{min, \kappa } ,$ is the minimum Lennard-Jones potential, $\sigma _{min, \kappa } ,$ is the molecular separation at which the potential is minimized, and $M_{\kappa }$ is the molecular mass. Likewise, nondimensional experimental temperatures are scaled using $T_{scale,\kappa } = \epsilon _{min, \kappa } / k_B ,$ where $k_B$ is Boltzmann’s constant. (b) The minimum temperature, $T_{min, \kappa } ,$ at which each specie viscosity was measured corresponds approximately to the melting temperature (at atmospheric pressure) for that specie. c) The maximum measurement temperature for each specie, $T_{max, \kappa } ,$ in all six cases, exceeds the specie’s atmospheric boiling point. Thus, the viscosities shown exceed the range of temperatures over which each specie is in the liquid state. Sources for the experimental viscosity data shown are as follows: $\mathrm {N_2}$^55^; $\mathrm {O_2}$^55^. For a discussion of estimated experimental uncertainties, please see the text.

Theoretical and experimental, temperature dependent dimensionless viscosities for the six simple liquids considered here are compared in Figs. 1 and 2. The comparisons lend significant support to our central argument: Decaying liquid viscosity in simple liquids reflects decreased electron screening of the positive nucleus. A similar mechanism may underlie temperature-dependent decay in surface tension coefficients in simple liquids, and may also play a role in viscosity and surface tension variations in, e.g., polar and ionic liquids.

In closing this section, we cite the sources of the experimental viscosity data presented in Figs. 1 and 2, as well as estimated uncertainties, where available. Regarding the latter^56^, reviews and correlates the data available through 2004, for liquid Ar, $\mathrm {N_2} ,$ and $\mathrm {O_2} .$ Using their temperature and pressure-dependent correlations for each specie, they define the *average absolute deviation*, AAD, of any given data set from the correlation, by the (schematic) relationship: $\mathrm {AAD} = | \left( X_{data} - X_{calc} \right) | / X_{calc} .$ Thus, their estimated AAD’s provide reasonable estimates for the uncertainty in any given experiment. References and estimated uncertainties for the seven sets of viscosity data shown in Figs. 1 and 2 are as folows: Ne^51^; uncertainty not reported; Ar^52^, AAD = 0.92 %; Kr^53^, uncertainty not reported; Xe^54^, uncertainty = 0.5 %; $\mathrm {N_2}$^55^, AAD = 8.48 %; $\mathrm {O_2}$^55^, AAD = 10.2 % ; $\mathrm {CH_4}$^57^, uncertainty not reported.

## Phonons and self-diffusion

In pursuing our objective of developing a picture of single molecule dynamics, within the framework of Langevin’s equation (5), we confront two additional, connected questions: a) What is the physical origin of the random force. $\mathbf {F_R} \left( t \right)$? Typically, $\mathbf {F_R} \left( t \right)$ is treated as a statistical entity, endowed with physically reasonable statistical properties^35,36^. In liquid-state dynamics problems, this mathematical approach reflects our poor physical understanding of $\mathbf {F_R} \left( t \right) .$ b) What is the origin of self-diffusion, i.e., the random, thermally-driven motion of individual molecules though a liquid? Since $\mathbf {F_R} \left( t \right)$ drives self-diffusion, answering either question offers insight into both.

There are two significant experimental clues: a) A series of experiments, carried out in the 1970’s^38^, measured the self-diffusion coefficient, $D_s = D_s \left( T, P \right) ,$ in liquid Ar, Kr, and Xe, over a range of temperatures and a series of fixed pressures, and lead to a (dimensionless) correlation of the following form:

15 $$\begin{aligned} D_s^* \left( T^* , P^* \right) = 1.1 \exp { \left( 0.16 P^* \right) } \exp { \left[ - \frac{2.39 + 0.23 P^*}{T^*} \right] } \end{aligned}$$

where $D_s^* = D_s / \sqrt{ \epsilon \sigma ^2 / M} ,$ $T^* = T / \left( \epsilon /k_B \right) ,$ and $P^*= P / \left( \epsilon / \sigma ^ 3 \right) .$ b) More recently, Bolmatov, Brazhkin and Trachenko^39,40^ presented strong evidence that temperature-dependent specific heats, in a large family of liquids, reflect existence of dominant, solid-state-like, equilibrium phonon modes.

Consider the solid-like dynamics of N-molecule liquid-state systems, over time scales ranging from the Frenkel to the dispersion scales, $\tau _{F} = 2 \pi / \omega _F = O \left( 10^{-14} \ \mathrm {s} \right)$ to $\tau _d = 2 \pi / \omega _d = O \left( 10^{-16} \ \mathrm {s} \right) .$ Using a normal mode analysis - as in solid-state systems - under the assumption that individual molecular oscillations remain small enough to approximate intermolecular potentials as quadratic in the oscillation amplitude, 3N independent, vibrational, i.e., phonon modes are determined. The principal limitation of this model centers on ignoring the random hops of individual molecules. However, based on two separate arguments and results presented below, it appears that molecular hopping, over the spectral range $\omega _F< \omega < \omega _{d} ,$ is limited to a narrow, low-frequency band of frequencies near the solid state limit: $\omega \approx \omega _F .$

On time scales on the order of, and shorter than $\tau _F ,$ and in the absence of single-molecule-scale external perturbations - like those produced by short wavelength neutron scattering beams - individual nuclei undergo small displacements, on the order of a small fraction of $\sigma .$ Under these conditions, and in terms of the Langevin model—see Table 2—the friction force can be neglected and the dynamics of individual molecules modeled using:

16 $$\begin{aligned} M \frac{d {\mathbf {v}}}{d t} = -M \sum _{i=1} \omega _i^2 \int _{0}^t \tilde{{\mathbf {v}}}_i \left( t , \tau \right) d \tau \end{aligned}$$

This equation states that on these time scales, individual molecules are subject to the summed effect of all phonon modes extant over the spectrum, $\omega _F \lesssim \omega \lesssim \omega _d .$ Specifically, the phonon mode having frequency $\omega _i ,$ induces an instantaneous nuclear velocity $\tilde{{\mathbf {v}}}_i \left( \tau , \omega _i \right) ,$ which, in turn, produces a nuclear displacement - over the small time interval $\left[ 0,t \right]$ - of $\Delta {\mathbf {x}}_i \left( \tau , \omega _i \right) = \int _0^t \tilde{{\mathbf {v}}}_i \left( \tau , \omega _i \right) d \tau .$ Since phonon modes are independent, the small displacements, $\Delta {\mathbf {x}}_i \left( \tau , \omega _i \right) ,$ are likewise. Thus, since $M \omega _i^2 \Delta {\mathbf {x}}_i \left( t \right)$ corresponds to the $i^{th}$ instantaneous spring force on the molecule, the sum of the random phonon-induced forces corresponds to the right side of (16).

### Derivation of the self-diffusion coefficient, $D_s$

From Note f) in the final section, on the solid-state-like time scale, $\tau _d \lesssim t \lesssim \tau _F ,$ the equation describing nuclear motion, driven by the $j^{th}$ phonon mode, is given by:

17 $$\begin{aligned} \frac{ d {\tilde{\mathbf {v}}_j} \left( t; \omega _j \right) }{dt} = - \omega _j^2 \int _0^t {\tilde{\mathbf {v}}_j} \left( t' ; \omega _j \right) dt' \approx - \omega _j^2 {\tilde{\mathbf {v}}_j} \left( t; \omega _j \ \right) t \end{aligned}$$

Solving this leads to

18 $$\begin{aligned} {\tilde{\mathbf {v}}_j} \left( t ; \omega _j \right) = {\tilde{\mathbf {v}}_j} \left( 0; \omega _j \right) \exp \left[ \frac{ - \omega _j^2 t^2}{2} \right] \end{aligned}$$

Thus, the instantaneous velocity of the nucleus corresponds to the superposition of phonon-induced velocity contributions:

19 $$\begin{aligned} {\mathbf {v}} \left( t \right) = \sum _{j} {\tilde{\mathbf {v}}_j} \left( t, \omega _j \right) \end{aligned}$$

so that the dot product, ${\mathbf {v}} \left( t \right) \cdot {\mathbf {v}} \left( 0 \right) ,$ is given by:

20 $$\begin{aligned} {\mathbf {v}} \left( t \right) \cdot {\mathbf {v}} \left( 0 \right) = \sum _{j} {\tilde{\mathbf {v}}_j} \left( t, \omega _j \right) \cdot \sum _{i} {\tilde{\mathbf {v}}_i} \left( 0, \omega _i \right) \end{aligned}$$

Due to the independence of phonon modes, and in light of (18),

21 $$\begin{aligned} \langle {\mathbf {v}} \left( t \right) \cdot {\mathbf {v}} \left( 0 \right) \rangle = \langle \sum _{j} {{\tilde{v}}^2_j} \left( 0; \omega _j \right) \exp \left[ \frac{ - \omega _j^2 t^2}{2} \right] \rangle \end{aligned}$$

where $$ {{\tilde{v}}^2_j} \left( 0; \omega _j \right) = {\tilde{\mathbf {v}}_j} \left( 0, \omega _j \right) \cdot {\tilde{\mathbf {v}}_j} \left( 0, \omega _j \right) .$$

The self-diffusion coefficient, $$ D_s ,$$ is given by the integrated velocity autocorrelation function:

22 $$\begin{aligned} D_s = \int _0^{\infty } \langle {\mathbf {v}} \left( t \right) \cdot {\mathbf {v}} \left( 0 \right) \rangle \ dt \end{aligned}$$

or

23 $$\begin{aligned} D_s = \int _0^{\infty } \Big \langle \sum _j {{\tilde{v}}^2_j} \left( 0; \omega _j \right) \exp \left[ \frac{ - \omega _j^2 t^2}{2} \right] \Big \rangle \ dt \end{aligned}$$

Integrating then gives:

24 $$\begin{aligned} D_s = \sqrt{\frac{\pi }{2}} \Big \langle \sum _j \frac{1}{\omega _j} {{\tilde{v}}^2_j} \left( 0; \omega _j \right) \Big \rangle \end{aligned}$$

In order to evaluate the equilibrium average: (i) recall that within a given volume, *V*,  the average number of phonons having frequency $\omega$ is given by^58,59^

25 $$\begin{aligned} \langle n_{\omega } \rangle = \frac{1}{\exp {\beta \hslash \omega } -1} \end{aligned}$$

(ii) at any location in *V*,  assume that the wave vector associated with each mode, over the ensemble, is isotropically oriented, and (iii) due to the nominally continuous distribution of modes, move to a continuum representation of the average in (24):

26 $$\begin{aligned} D_s = \frac{1}{3} \sqrt{\frac{\pi }{2}} \int _{\omega _F}^{\omega _d} \frac{ g \left( \omega \right) }{\exp {\beta \hslash \omega } -1} \frac{{\tilde{v}}^2 \left( 0 ; \omega \right) }{ \omega } \ d \omega \end{aligned}$$

where $g \left( \omega \right)$ is the density of modes *driving self-diffusion*. Finally, in order to arrive at a theoretical $D_s$ having the same generic structure as the empirical $D_s$ in (15), we assume that the density of modes driving self-diffusion is clustered around a critical frequency, $\omega _c :$

27 $$\begin{aligned} g \left( \omega \right) = \delta \left( \omega - \omega _c \right) \end{aligned}$$

As described below, this assumption leads to a detailed, physically consistent explanation of phonon-driven self-diffusion in simple, nonpolar, nonmetallic liquids.

Using (27) in (26), approximating $\exp {\beta \hslash \omega } -1$ as $\exp {\beta \hslash \omega } ,$ and nondimensionalizing using $D_s^* = D_s / \sqrt{ \epsilon \sigma ^2 / M} ,$ $T^* = T / \left( \epsilon /k_B \right) ,$ and $P^*= P / \left( \epsilon / \sigma ^ 3 \right) ,$ finally leads to:

28 $$\begin{aligned} D_{s, \alpha }^* \left( T^* , P^* \right) = \frac{ \langle {\tilde{v}}_{c,\alpha }^2 \rangle \sqrt{ \pi /2} }{ \omega _{c, \alpha } \left( \frac{ \epsilon _{\alpha } \sigma _{\alpha }^2}{M_{\alpha }} \right) ^{1/2} } \exp { \left[ -\frac{\hslash \omega _{c, \alpha } / \epsilon _{\alpha }}{T_{\alpha }^*} \right] } \end{aligned}$$

where $\alpha$ denotes either Ar, Kr, or Xe, and where two undetermined, pressure-dependent parameters, $\langle {\tilde{v}}^2 \left( 0, \omega _c \right) \rangle$ and $\omega _{c, \alpha } ,$ appear. The first,

29 $$\begin{aligned} \langle {\tilde{v}}_{c,\alpha }^2 \rangle = \langle {\tilde{v}}^2 \left( 0, \omega _c ; P^* \right) \rangle \end{aligned}$$

is the phonon-induced, ensemble averaged, pressure-dependent, squared velocity of the molecule, evaluated at the critical phonon frequency,

30 $$\begin{aligned} \omega _{c,\alpha } = \omega _{c,\alpha } \left( P^* \right) \end{aligned}$$

where $\omega _c$ is the frequency that induces significant, single-atom-scale, random jumps, i.e., self-diffusion. The physical meaning of these parameters is explored in the next section. Note, approximating $\exp {\beta \hslash \omega } -1$ as $\exp {\beta \hslash \omega } ,$ - again, introduced in order to arrive at a theoretical $D_s$ having the same form as (15) - is based on the fact that, in liquid Ar, Kr, and Xe, $\exp {\beta \hslash \omega } = O \left( 10 \right) .$

### Phonon-induced hopping speeds and critical frequencies; comparisons with experimental self-diffusion coefficients

In order to determine $\langle {\tilde{v}}_{c,\alpha }^2 \rangle$ and $\omega _{c,\alpha } ,$ we use the experimental correlation^38^ (15), leading to

31 $$\begin{aligned} \omega _{c, \alpha } \left( P^* \right) = \left( 2.39 + 0.23 P^* \right) \epsilon _{\alpha } / \hslash \end{aligned}$$

and

32 $$\begin{aligned} \langle {\tilde{v}}_{c,\alpha }^2 \left( P^* \right) \rangle = 1.1 \cdot \omega _{c, \alpha } \left( \frac{ \epsilon _{\alpha } \sigma _{\alpha }^2}{M_{\alpha }} \right) ^{1/2} \sqrt{2/ \pi } \exp { \left( 0.16 \cdot P^* \right) } \end{aligned}$$

**Figure 3:**
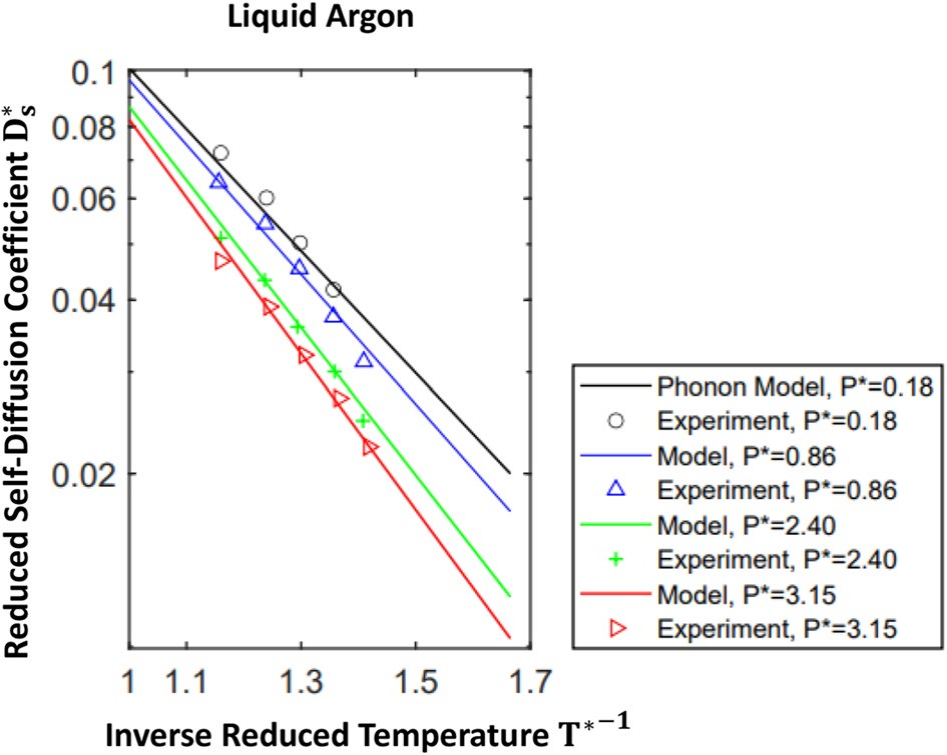
Temperature-dependent self-diffusion coefficient of liquid argon. Over the spectrum of frequencies available to a liquid state system, $$ 0 \le \omega \lesssim \omega _d , $$ the phonon self-diffusion model: (i) idealizes the band from the Frenkel frequency, $$ \omega _F = \mathrm {O} \left( 10^{14} \ \mathrm {s^{-1}} \right) , $$ to the dispersion frequency, $$ \omega _d = \mathrm {O} \left( 10^{16} \ \mathrm {s^{-1}} \right) , $$ as corresponding to solid-state-like dynamics, (ii) assumes that on $$ \omega _F \lesssim \omega \lesssim \omega _d , $$ individual molecules undergo small amplitude, harmonic vibrations about fixed positions, and (iii) thus allows a normal mode analysis of the solid-like dynamics. In order to capture the observed temperature dependence of $$ D_s^* $$^38^, it is necessary to assume that the band of phonon frequencies driving self-diffusive, single molecule random hops is concentrated near the low end of the solid state spectrum, $$ \omega \approx \omega _F,$$ idealized as a delta function in (27). The nondimensional definition of $$ D_s^* $$ is given following (15). The experimental self-diffusion data was reported in^38^; experimental uncertainty was estimated to be less than 5 %^38,61^.

**Figure 4:**
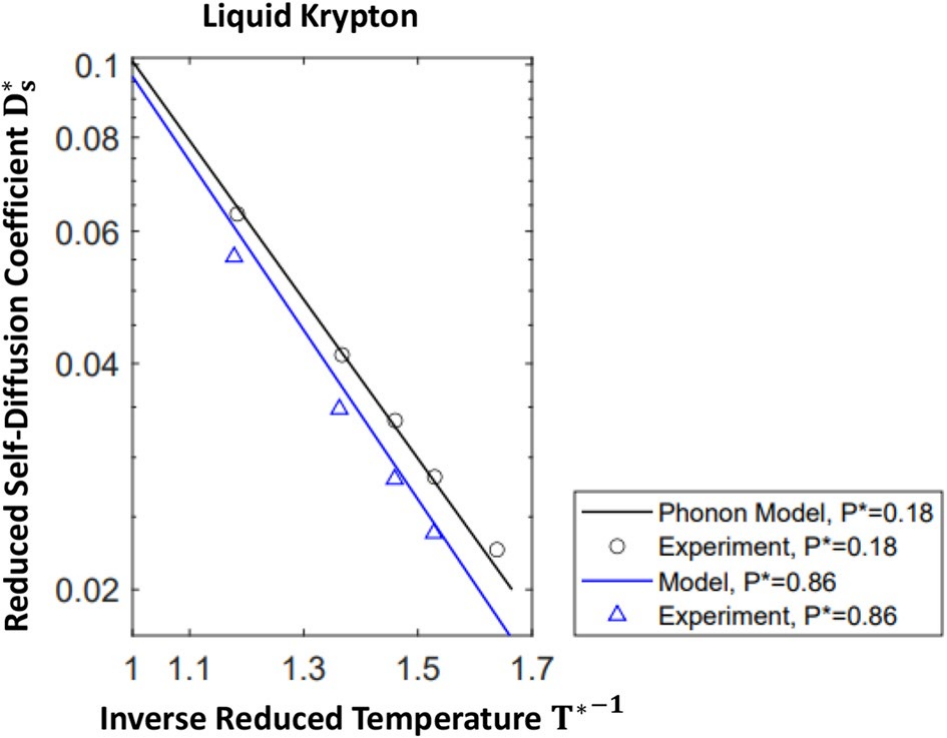
Temperature-dependent self-diffusion coefficient of liquid krypton. See the caption to Fig. 3 for a description of the phonon model of self-diffusion. The experimental self-diffusion data was reported in^38^; experimental uncertainty was estimated to be less than 5 %^38,61^.

**Figure 5:**
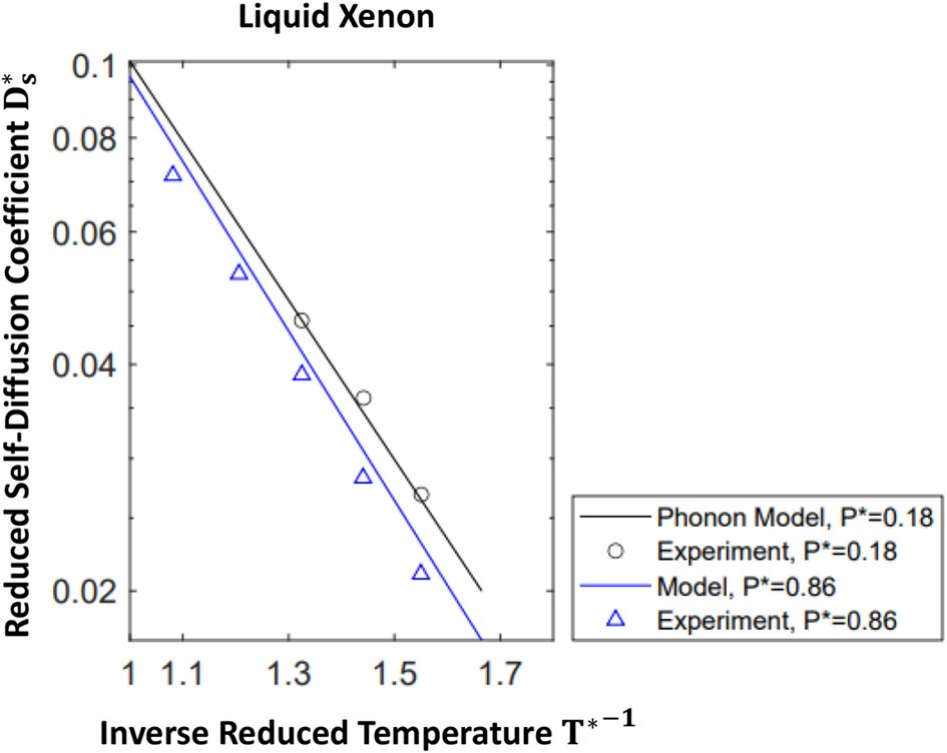
Self-diffusion coefficient for liquid Xenon. See the caption to Fig. 3 for a description of the phonon model of self-diffusion. The experimental self-diffusion data was reported in^38^; experimental uncertainty was estimated to be less than 5 %^38,61^.

Comparisons of temperature- and pressure-dependent self-diffusion coefficients, $$ D_s^* \left( T^*, P^* \right) , $$ predicted by the phonon-based model, (28), with experimental measurements^38^ in liquid Ar, Kr, and Xe, are shown in Figs. 3, 4, and 5. Pressure-dependent magnitudes of the critical phonon frequency, $$ \omega _{c, \alpha },$$ driving self-diffusion, and the root mean square atomic speed, $$ \sqrt{ \langle {\tilde{v}}_{c,\alpha }^2 \left( P^* \right) \rangle } , $$ induced by these critical phonons, are listed in Table 1. Note that the slight apparent offset between the theoretical correlations and measured self-diffusion coefficients, observed at $$P* =0.86 ,$$ in Figs. 4 and 5, reflects nondimenisonalization of *P*,  *T*,  and $$ D_s , $$ by the same set of experimental $$ \epsilon '\mathrm {s} $$ and $$ \sigma '\mathrm {s} $$^41^ used to nondimensionalize temperatures and viscosities in Figs. 1 and 2. See^38^ for references to the sources of the $$ \epsilon '\mathrm {s} $$ and $$ \sigma '\mathrm {s} $$ used in their study, and note that ca. 1962, variations in experimentally observed magnitudes of these parameters was on the order of 3 %^38^.

In the next section, we present an alternative derivation of the self-diffusion coefficient which again assumes that phonons underlie self-diffusion, but which models the high frequency liquid state, $$ \omega _F \lesssim \omega \lesssim \omega _d , $$ as a bond-free Einstein solid in which all nuclei vibrate - in cages of surrounding molecules—at or near a fixed (Einstein) frequency, $$ \Omega _o . $$ This argument leads to a $$ D_s^* ,$$ (40), having the same generic form as (28), but derived from a significantly different physical viewpoint.

As a preliminary consistency check on the general picture of phonon-driven self-diffusion, leading to the semi-empirical expressions for the characteristic hopping frequency, $\omega _{c,\alpha } ,$ and speed of hopping molecules in $\langle {\tilde{v}}_{c,\alpha }^2 \rangle ,$ (31) and (32), respectively, we highlight the following points:

(a) For small to moderate reduced pressures, $P^* = O \left( 1 \right) ,$ (32) leads to the following approximate equality:

33 $$\begin{aligned} \frac{\langle {\tilde{v}}_{c,\alpha }^2 \rangle }{ \omega _{c, \alpha }} \approx \left( \frac{ \epsilon _{\alpha } \sigma _{\alpha }^2}{M_{\alpha }} \right) ^{1/2} \left( = D_{so, \alpha } \right) \end{aligned}$$

where $$ D_{so, \alpha } $$ is the scale of the self-diffusion coefficient. By contrast, as a check on the steps leading from insertion of the integrated single molecule dynamics equation, (16), to the expression for $$ D_s , $$ written in the form:

34 $$\begin{aligned} D_s = \int _0^{\infty } \left[ \int _{\omega _c}^{\omega _D} \frac{g \left( \omega \right) }{ \left( \exp {\beta \hslash \omega }-1 \right) } f \left( \omega \right) d \omega \right] dt \end{aligned}$$

the left side of (33) can be obtained by starting with the definition, $D_{s, \alpha } = \int _0^{\infty } \langle {\mathbf {v}} \left( t' \right) \cdot {\mathbf {v}} \left( 0 \right) \rangle dt' ,$ and replacing the upper limit with the characteristic time scale for single-molecule hops, $\tau _{hop, \alpha } = \omega _{c, \alpha }^{-1} ,$ where the latter captures the assumed delta-function density of hop-inducing phonons near $\omega _{c, \alpha } ,$ (27). This leads to $D_{s, \alpha } = \langle {\mathbf {v}} \left( 0 \right) \cdot {\mathbf {v}} \left( 0 \right) \rangle \cdot \omega _{c , \alpha }^{-1} = \langle {\tilde{v}}_{c, \alpha }^2 \rangle \cdot \omega _{c , \alpha }^{-1} .$

(b) For all three liquids, Ar, Kr and Xe, estimated critical phonon frequencies, $\omega _{c, \alpha }$—which we interpret as the characteristic hopping frequency—lie well within the range of frequencies, $\omega _{d, \alpha }> \omega _{c, \alpha } > \omega _{F, \alpha } ,$ where these liquids maintain solid-like properties^40^. Estimated ${\omega _{c, \alpha }}'s$ are approximately six times higher than estimated Frenkel frequencies^39,40^, $\omega _{c, \alpha } \approx 6 \omega _{F, \alpha } = 12 \pi G_{\infty , \alpha } / \mu _{\alpha } ,$ and approximately two orders of magnitude smaller than characteristic dispersion frequencies, $\omega _{d, \alpha } ,$ where $G_{\infty , \alpha }$ and $\nu _{\alpha }$ are, respectively, the high-frequency shear modulus^22,39,40^ and dynamic viscosity of specie $\alpha ,$ and where magnitudes of $G_{\infty , \alpha }$ are obtained from^39^, and magnitudes of $\nu _{\alpha }$ are given in Supplement 4.

(c) Magnitudes of molecular hopping speeds, $\sqrt{ \langle {\tilde{v}}_{c,\alpha }^2 \rangle },$ exceed, by roughly a factor of two, both the longitudinal liquid-state sound speed^60^, $a_{liq} = \sqrt{ K / \rho } ,$ and the slightly faster longitudinal solid-state sound speed, $a_{solid} = \sqrt{ a_L^2 + 4/3 a_S } ,$ where *K* is the bulk modulus and $a_S = \sqrt{ G_{\infty } / \rho }$ is the shear (transverse) wave speed. Thus, average atomic hopping speeds are well in excess of characteristic liquid- and solid-state sound speeds. Equivalently, from (31), the hop-inducing phonon energy, $\hslash \omega _{c, \alpha } \left( P^* \right) ,$ is approximately twice the intermolecular energy scale, $\epsilon _{\alpha } ,$ and increases (linearly) with pressure.

**Table 1:** According to the proposed model of phonon-driven self-diffusion, over the portion of the frequency spectrum where liquid dynamics are solid-like, $\omega _F \lesssim \omega \lesssim \omega _d ,$ the instantaneous velocity of any given nucleus is determined by superposition of 3*N* independent, phonon-induced velocity contributions: ${\mathbf {v}} \left( t \right) = \sum _{j} {\tilde{\mathbf {v}}_j} \left( t, \omega _j \right)$.

Phonon Induced Hopping Speed and Critical Frequency								
Reduced Pressure	P*=0.18		P*=0.86		P*=2.4		P*=3.15	
Specie	$\sqrt{\langle {v}_{c}^2 \rangle }$	$\omega _c \times 10^{13}$	$\sqrt{\langle {v}_{c}^2 \rangle }$	$\omega _c \times 10^{13}$	$\sqrt{{v}_{c}^2 }$	$\omega _c \times 10^{13}$	$\sqrt{{v}_{c}^2 }$	$\omega _c \times 10^{13}$
	(m/s)	$\mathrm {s^{-1}}$	(m/s)	$\mathrm { s^{-1}}$	(m/s)	$\mathrm {s^{-1}}$	(m/s)	$\mathrm {s^{-1}}$
Ar	1340	3.70	1470	3.94	1780	4.48	1950	4.74
Kr	1660	6.06	1813	6.45	NM		NM	
Xe	1814	7.30	1980	7.77	NM		NM	

Based on this correspondence and the assumption, (27), that self-diffusive hops are produced by a narrow band of phonon modes centered near a critical frequency, $\omega _c ,$ we identify $\sqrt{\langle {v}_{c}^2 \rangle }$ as the hopping speed. NM = not measured.

### A second picture of self-diffusion: the high-frequency liquid state corresponds to a bond-free Einstein solid

The argument in the two previous sections leads to semi-empirical expressions for a characteristic hopping frequency, $\omega _{c,\alpha } ,$ and the speed of hopping molecules in $\langle {\tilde{v}}_{c,\alpha }^2 \rangle ,$ (31) and (32). In order to reinforce and broaden this argument, we outline a second derivation of $D_s$ that again assumes that self-diffusion is driven by liquid-state phonon modes. The second argument leads to an alternative, but not inconsistent picture of self-diffusion: (i) High frequency molecular dynamics in nonpolar liquids, over $\omega _F \lesssim \omega \lesssim \omega _d ,$ can be modeled as those in a bond-free Einstein solid, in which each molecule vibrates as an independent oscillator, trapped in a cage of surrounding, nominally fixed molecules^62^. (ii) As described below, self-diffusive hops of individual, initially trapped molecules takes place when the molecule is pushed sufficiently far into a short-lived repulsive state with one of its neighbors.

Four observations provide the basis for this second picture. First, the velocity autocorrelation function (VACF) in Lennard-Jones liquids, e.g., noble liquids, is largely determined by the repulsive part of the LJ potential^63^. Second, consistent with the fact that repulsive states are short-lived, the short time scale normalized VACF for liquid argon, $0 \le t \lesssim \tau _c ,$ is well-captured by a quadratic short-time expansion^22,64^:

35 $$\begin{aligned} \psi \left( t \right) = 1 - \frac{\Omega _o^2 t^2}{2!} + O \left( t^4 \right) \end{aligned}$$

where

36 $$\begin{aligned} \Omega _o^2 = \frac{\langle {\dot{\mathbf {v}}} \left( 0 \right) \cdot {\dot{\mathbf {v}}} \left( 0 \right) \rangle }{\langle {\mathbf {v}} \left( 0 \right) \cdot {\mathbf {v}} \left( 0 \right) \rangle } \end{aligned}$$

is the liquid-state Einstein frequency, and where $$ \psi \left( t \right) = \langle {\mathbf {v}} \left( t \right) \cdot {\mathbf {v}} \left( 0 \right) \rangle \left[ \langle {\mathbf {v}} \left( 0 \right) \cdot {\mathbf {v}} \left( 0 \right) \rangle \right] ^{-1} . $$ Third, over the liquid-state frequency spectrum, $$ 0 \le \omega \lesssim \omega _F , $$ $$ \Omega _o $$ can be interpreted as the characteristic oscillation frequency of individual liquid state molecules, trapped in a cage of surrounding molecules, all held in their equilibrium positions^34^. Importantly, while characteristic $$ \Omega _o ' \mathrm {s} $$ have been calculated in Lennard-Jones liquids^30^, these assume Maxwell-Boltzmann (MB) (kinetic) energy distributions, appropriate to the liquid state portion of the frequency spectrum. Apparently, similar calculations have not been reported over the solid state end of the spectrum, $$ \omega _d \lesssim \omega \lesssim \omega _F ,$$ where Bose-Einstein statistics apply. Significantly, as shown immediately below, use of the liquid-appropriate MB distribution in evaluating the averages in (36) leads to incorrect temperature scaling in $$ D_s , $$ whereas the solid-appropriate Bose-Einstein (BE) distribution provides the correct scaling. Fourth, assuming that wave speeds, $$ v_i = \omega _i / k_i , \ i =1, 2,\ldots , 3N , $$ of the 3*N* independent phonon modes are of the order of the longitudinal liquid-state or solid-state sound speed, $$ a_{liq} $$ or $$ a_{solid} , $$ respectively (where again, $$ a_{liq} \approx a_{solid} $$ ), we find that over the spectrum of available solid-state-like phonon frequencies, $$ \omega _F \lesssim \omega _i \lesssim \omega _d , $$ phonon wavelengths, $$ \lambda _i, $$ are all shorter than approximately $$ 2 \sigma /3. $$ Thus, in contrast to, e.g., crystalline solids, collective, multi-molecule oscillations are nonexistent.

To begin the second derivation, truncate the expansion in (35) at the quadratic term, and approximate the quadratic as an exponential, $1 - \frac{\Omega _{op}^2 t^2}{2!} = \exp \left( - \Omega _{op}^2 t^2 / 2 \right) ,$ where $\Omega _{op}$ denotes the Einstein frequency determined by use of the BE distribution in (36). In order to estimate the error in this approximation, first inspect approximate mean frequency-dependent phonon occupation numbers, $\langle n \left( \omega \right) \rangle = \left[ e^{\hslash \omega / k_B T } - 1 \right] ^{-1} :$ Doing so, we find that at a characteristic temperature of order $10^2 \ \mathrm {K} ,$ $\langle n \left( \omega _F \right) \rangle \sim 10^{-5} ,$ $\langle n \left( 10 \omega _F \right) \rangle \sim 10^{-44} ,$ and $\langle n \left( \omega _d \right) \rangle \sim \langle n \left( 10^2 \omega _F \right) \rangle \sim e^{-1000} \rightarrow 0 .$ Thus, consistent with the first argument leading to (28) above, where it is assumed that the portion of the phonon spectrum driving self-diffusion lies in the neighborhood of $\omega _F ,$ we assume that the solid state-like Einstein frequency, $\Omega _{op} ,$ is on the order of $\omega _F .$ In turn, over phonon time scales, $\tau _d \lesssim t \lesssim \tau _F ,$ the relative error, $2 \Omega _{op}^2 t^2 / 4! ,$ in replacing the quadratic in (35) with an exponential is, at most, on the order of 10 %.

Given the exponential approximation for the short-time VACF in (35), $D_s$ can now be expressed as

37 $$\begin{aligned} D_s = \langle {\mathbf {v}} \left( 0 \right) \cdot {\mathbf {v}} \left( 0 \right) \rangle \int _0^{\infty } \exp \left( \frac{- \Omega _{op}^2 t^2}{2} \right) dt = \sqrt{ \frac{\pi }{2}} \frac{1}{\Omega _{op}} \langle {\mathbf {v}} \left( 0 \right) \cdot {\mathbf {v}} \left( 0 \right) \rangle \end{aligned}$$

Importantly, if we assume that liquid-state dynamics determines self-diffusion, then $$ \langle {\mathbf {v}} \left( 0 \right) \cdot {\mathbf {v}} \left( 0 \right) \rangle = 3 k_b T /M, $$ and, as noted in^22^ ,$$ \Omega _{op} \propto T^{1/4} ;$$ in this case, $$ D_s \propto T^{3/4} , $$ which is clearly inconsistent with the experimentally observed^38^ exponential dependence, $$ D_s \propto \exp \left( -A^2 / T \right) ,$$ shown, for example, in (28) (where $$ A^2 $$ is a positive real number). By contrast, as shown in the first argument leading to (28) above, solid-like phonon dynamics do provide the appropriate scaling. Thus, repeating the analysis from (17) to (21), and setting $$ t=0 ,$$ we obtain

38 $$\begin{aligned} \langle {\mathbf {v}} \left( 0 \right) \cdot {\mathbf {v}} \left( 0 \right) \rangle = \langle \sum _{j} {{\tilde{v}}^2_j} \left( 0; \omega _j \right) \rangle \end{aligned}$$

The fourth observation above, combined with the fact that $\beta \hslash \omega$ is large over $\omega _F \lesssim \omega \lesssim \omega _d ,$ suggests that the Einstein model of solid state phonons^58,62^ - which captures incoherent solid state atomic dynamics - is an appropriate model for liquid state phonons. Thus, in order to evaluate $\langle {\mathbf {v}} \left( 0 \right) \cdot {\mathbf {v}} \left( 0 \right) \rangle ,$ we repeat the integral in (26):

39 $$\begin{aligned} \langle {\mathbf {v}} \left( 0 \right) \cdot {\mathbf {v}} \left( 0 \right) \rangle = \int _{\omega _F}^{\omega _d} \frac{ g \left( \omega \right) }{\exp {\beta \hslash \omega } -1} {\tilde{v}}^2 \left( 0 ; \omega \right) \ d \omega \end{aligned}$$

where now, the density of (short wavelength, non-collective) phonon states, $g \left( \omega \right) ,$ has the same mathematical definition, $g \left( \omega \right) = \delta \left( \omega - \Omega _{op} \right) ,$ but a qualitatively distinct interpretation: Over the spectrum of exclusively short wavelength, non-collective phonon modes available to an $N-\mathrm {molecule}$ liquid state system, $g \left( \omega \right)$ *captures an assumed, nominally fixed density of phonon modes driving self-diffusive hops of individual molecules.* Assuming that the density of *all phonon states* - which encompasses the narrow band driving self-diffusion - is reasonably well modeled by the Debye model^58^, or more generally, by any other solid state phonon density model, then both interpretations of $g \left( \omega \right) ,$ here and above, are completely equivalent.

Again, since $$ \beta \hslash \omega>> 1 , $$ we can drop the negative one in the denominator in (39), carry out the integral, insert the result in (37), highlight, based on the measurements in^38^, the temperature and pressure-dependence of $$ D_s , $$ and nondimenisionalize as in the first derivation, to obtain a second (dimensionless) version of $$ D_s :$$

40 $$\begin{aligned} D_{s, \alpha }^* \left( T^* , P^* \right) = \frac{1}{3} \frac{ \langle {\tilde{v}}_{c,\alpha }^2 \rangle \sqrt{ \pi /2} }{ \Omega _{op, \alpha } \left( \frac{ \epsilon _{\alpha } \sigma _{\alpha }^2}{M_{\alpha }} \right) ^{1/2} } \exp { \left[ -\frac{\hslash \Omega _{op, \alpha } / \epsilon _{\alpha }}{T_{\alpha }^*} \right] } \end{aligned}$$

where the ’critical phonon frequency’, $\omega _c ,$ of phonon-driven molecular hopping, is now interpreted as the solid state (as opposed to liquid state) Einstein frequency, and where all other terms have the same definitions as before.

Importantly, using two physically distinct arguments, we have derived the same expression for the diffusion coefficient, which, in both cases, has the appropriate $\exp \left( -A^2 /T \right)$ temperature scaling, and which - as described above and below - provides a physically consistent picture of the molecular scale mechanisms driving self-diffusion.

## Electron cloud compression, single molecule hopping, and shear-induced viscosity generation

The argument leading to the theoretical expression for dynamic viscosity, (14), is built on the idea that, in non-metallic, nonpolar liquids, polarizability - the susceptibility of a molecule’s electron cloud to distort under the action of an external electric field - plays a dominant role in determining the friction force acting on the molecule. Although not apparent in the arguments leading to theoretical self-diffusion coefficients in (28) and (40), the notion that electron cloud distortion also plays a dominant role here emerges from three observations. First, configuration-averaged instantaneous normal mode (INM) spectra in solid- and liquid-state systems show that, at any instant, a significant fraction of interacting molecular pairs in liquids are in a state of mutual repulsion; in corresponding solids, only a small fraction of pairs are in such states^65^. Second, as noted above, the VACF in Lennard-Jones, e.g., noble liquids, is largely determined by the repulsive part of the LJ potential^63^. Third, the argument leading to (40) exposes the notion that self-diffusive hops of single molecules are somehow connected to pre-hop vibration of the molecule, vibrating (at the solid-state Einstein frequency) within a cage of surrounding molecules. Together, these observations suggest that self-diffusive hops result from repulsion-inducing compression of both the trapped molecule’s electron cloud, as well as that of one or more of its neighbors. [Again, Note (i) in the final section indicates that pair-wise interactions are dominant.]

More generally, London dispersion forces comprise the *only* intermolecular force extant in non-metallic, nonpolar liquids, and these, in turn, are wholly determined by variations in polarizability^41^. Thus, building qualitative understanding of the essential connection between electron cloud distortion and single molecule viscous and self-diffusion forces represents an important task. Computational chemistry and biology^66^ offer a number of techniques for visualizing distortion and modification of electron distributions that accompany various intermolecular interactions^67,68^. Unfortunately, while dispersive interactions are ubiquitous and must be accommodated to accurately capture, e.g., solvation-induced changes in polyatomic molecular structure^68^, development of predictive dispersion interaction models remains an open problem^68^.

Visualization of electron cloud distortion accompanying self-diffusion and viscosity emergence in liquids, including that in nonmetallic, nonpolar liquids, apparently has not been undertaken. In order to build qualitative understanding of the essential connection between cloud distortion and these fundamental dynamical processes, we introduce two parameters, $$ x_{disp} $$ and $$ x_{diff} ,$$ that respectively serve as rough indicators of the specie-dependent electron cloud distortions that accompany self-diffusion and emergence of viscosity.

Considering first electron cloud distortion associated with self-diffusion, we imagine a trapped molecule, vibrating at or near the solid-state Einstein frequency, $\Omega _{op} \left( = \omega _c \right) ,$ in a cage of surrounding molecules. Again, as argued in the final section, Note i), on all time scales exceeding $O \left( \tau _d \right) ,$ pair-wise intermolecular collisions dominate 3-body and higher-order collisions. Writing Shrodinger’s equation for the trapped molecule, $i \hslash \psi _{, t} + \hslash ^2 \nabla ^2 / \left( 2 M \right) = V \psi ,$ estimating the scales of the two terms on the left side, where the time scale is determined by the Einstein (= the critical hopping) frequency, $t \sim \Omega _{op}^{-1} = \omega _{c}^{-1} ,$ we find that the length scale on which quantum uncertainty effects are important, $x_Q = x_{DeBroglie} \sim \sqrt{ \hslash / 2 M \omega } ,$ is small relative to the molecular diameter: $\sigma :$ $x_Q / \sigma \sim 10^{-2} .$ Thus, at least in the vicinity of $\omega \sim \Omega _{op} = \omega _{c},$ nuclear motion is classical.

**Figure 6:**
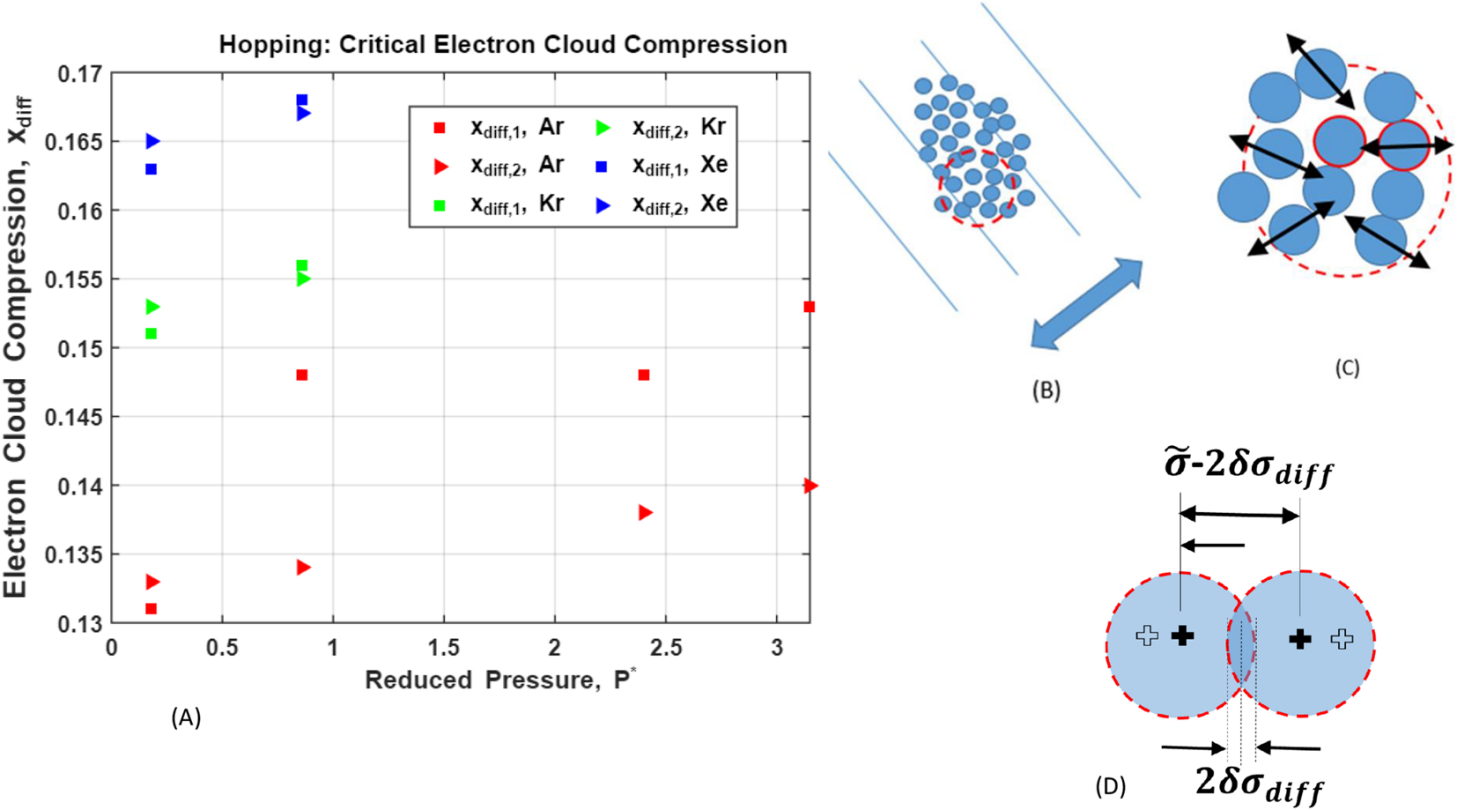
Two observations^63,65^ provide important clues concerning the mechanism driving self-diffusion in liquids: (i)^65^ shows that at any instant, a significant fraction of interacting molecular pairs in liquid Ar exist in a state of mutual repulsion, while in the corresponding solid, only a small fraction of pairs are in such states. (ii) The velocity autocorrelation function (VACF) in Lennard–Jones liquids, e.g., noble liquids, is largely determined by the repulsive part of the potential^63^. Viewed in terms of the proposed phonon models of self-diffusion, and given the dominance of pair-wise collisions, these observations suggest that the relatively large single-molecule kinetic energies required for hopping are supplied by collisional compression of adjacent electron clouds. Here, $$ x_{diff,1, 2} = \delta \sigma _{diff,1 ,2} / {\tilde{\sigma }} ,$$ are estimated relative compressions of individual clouds, and $$ {\tilde{\sigma }} = 2^{1/6} \sigma $$ is the intermolecular separation minimizing the LJ potential. See the text for derivations of $$ x_{diff,1} $$ and $$ x_{diff,2} , $$ as well as a short discussion of the gap between compression estimates observed for Ar. In order to contrast liquid state phonon modes, whose wavelengths are all smaller than or approximately equal to $$ 2 \sigma / 3 , $$ with collective, hydrodynamic modes that emerge on time scales exceeding $$ \tau _c = \mathrm {O}\left( 10^{-13} \ \mathrm {s} \right) $$ $$[$$ where solid-like dynamics take place on $$ \tau _d = \mathrm {O} \left( 10^{-16} \ \mathrm {s} \right) \lesssim t \lesssim \tau _F = \mathrm {O} \left( 10^{-14} \ \mathrm {s} \right) $$ $$ ] ,$$ (**B**) depicts a (short-wavelength, standing) sound wave (blue double arrow). Panel (**C**) depicts the jitter-like phonon oscillations that are superposed on slower hydrodynamic modes. Panel (**D**) depicts the electron cloud compression driving self-diffusion.

The apparent mechanism driving molecular hops is sketched in Fig. 6. Since nuclear motion on the $\tau _F$ time scale is classical, we can apply the classical version of conservation of energy to the interaction between a fixed target (cage) molecule, ${\mathscr {A}},$ and a colliding (trapped, vibrating) molecule, ${\mathscr {B}} .$ On approach toward ${\mathscr {A}},$ ${\mathscr {B}}$ is assumed to have sufficient (relative) kinetic energy and (relative) momentum to allow ${\mathscr {A}}$ and ${\mathscr {B}}$ to enter a repulsive state. Applying conservation of energy to ${\mathscr {B}} ,$ from the instant when maximum electron cloud compression occurs - and the relative velocity of ${\mathscr {B}}$ is ${\mathbf {0}}$ - to the instant when the intermolecular separation, $r_{\mathscr {AB}} ,$ equals the LJ potential minimizing separation, ${\tilde{\sigma }} = 2^{1/6} \sigma ,$ we obtain:

41 $$\begin{aligned} \frac{M}{2} \left[ \langle v_c^2 \rangle - \langle v_i^2 \rangle \right] \approx - \epsilon \int _{r_c}^{{\tilde{\sigma }}} \frac{\partial }{\partial r} \left( \frac{{\tilde{\sigma }}}{r} \right) ^{12} dr \end{aligned}$$

where the intermolecular potential is dominated by repulsion, and where the equation represents the ensemble average dynamics of $$ {\mathscr {A}} $$ and $$ {\mathscr {B}} $$ for a single collision. Using $$ \langle v_i^2 \rangle = 0 ,$$ as well as the relationship $$ \delta \sigma _{diff,1} = \left( {\tilde{\sigma }} - r_c \right) /2 ,$$ then leads to an estimate for the fractional electron cloud compression, $$ x_{diff,1} ,$$ that produces single molecule hops:

42 $$\begin{aligned} x_{diff,1} = \frac{\delta \sigma _{diff}}{{\tilde{\sigma }}} = \frac{ \left( {\tilde{\sigma }} - r_c \right) }{{\tilde{\sigma }}} \approx \frac{1}{2} \left[ 1 - \left( 1 + \frac{M \langle v_c^2 \rangle }{2 \epsilon } \right) ^{-1/12} \right] \end{aligned}$$

Resolving the relative motion of $$ {\mathscr {A}} $$ and $$ {\mathscr {B}} $$ into normal and tangential directions, the minimum nuclear separation between $$ {\mathscr {A}} $$ and $$ {\mathscr {B}} $$ corresponds to $$ r_c , $$ and $$ \delta \sigma _{diff} $$ represents the maximum inward displacement, in the normal direction, of each colliding nucleus. See Fig. 6, D. Note, that while the LJ model is well-suited to noble liquids, other pair-wise potentials are available;^69^ highlights other possibilities, as well as limitations of the LJ model. Note too, in order to avoid over-estimates of $$ \sqrt{ \langle v_c^2 \rangle } $$ (by a factor of $$ \sqrt{3} ) , $$ one-dimensional diffusion coefficients are used throughout. For simple isotropic liquids, these can be measured, for example, by measuring displacements of isotopes in a given direction^70^.

Assuming the validity of the simple energy conservation argument leading to (42), a second estimate of $$ \delta \sigma _{diff} ,$$ representing a direct connection between self-diffusion and electron cloud compression, can be derived. First, as described above, evaluation of (34), leads to $$ D_{s, \alpha } = \langle {\mathbf {v}} \left( 0 \right) \cdot {\mathbf {v}} \left( 0 \right) \rangle \cdot \omega _{c , \alpha }^{-1} = \langle {\tilde{v}}_{c, \alpha }^2 \rangle \cdot \omega _{c , \alpha }^{-1} . $$ Since again, $$ D_s $$ scales as $$ \sqrt{ \epsilon \sigma ^2 / M ,} $$ , then $$ \langle v_c^2 \rangle \sim \sqrt{ \epsilon \sigma ^2 / M ,} \omega _s . $$ Using this approximation in (42), defining the characteristic frequency of collective, thermally-driven nuclear oscillations as $$ \omega _{th} = \sqrt{ \epsilon / \left( M \sigma ^2 \right) } , $$ using the fact that, in liquid Ar, Kr, and Xe, $$ \omega _{th} = O \left( 10^{11} \mathrm {s^{-1}} \right) , $$ while $$ \omega _c = O \left( 10^{13} - 10^{14} \mathrm {s^{-1}} \right) , $$ and Taylor expanding the $$ \left( \cdot \right) ^{-1/12} $$ term in (42) for small $$ \omega _{th}/ \omega _c ,$$ we obtain a second estimate for, $$ x_{diff,1} ,$$ which we label as $$ x_{diff,2} :$$

43 $$\begin{aligned} x_{diff,2} \approx \frac{1}{2} \left[ 1 - \left( \frac{2 \omega _{th} }{\omega _c} \right) ^{1/12} \cdot \left[ 1 + \frac{1}{12} \left( \frac{2 \omega _{th}}{\omega _c} \right) \right] ^{-1} \right] \end{aligned}$$

Estimated, pressure-dependent magnitudes of $x_{diff,1}$ and $x_{diff,2}$ for Liquid Ar, Kr and Xe, are plotted in Fig. 6. Both estimates assume fast, repulsion-driven, collisionless acceleration of a single hopping molecule, initially trapped and oscillating in a cage of surrounding molecules. However, while both estimates also connect $\langle v_c^2 \rangle$ to the characteristic $D_s$ scale $\left( D_s \sim \sqrt{ \epsilon \sigma ^2 /M} \right) ,$ the second estimate alone incorporates the critical hopping frequency, $\omega _c .$ Thus, the consistency of both estimates lends support to the proposed picture of cloud-compression-driven molecular hopping, as well as further support for the proposed general picture of phonon-driven self-diffusion. Note that the approximate 10 % gap between compression estimates observed for Ar (for $P^* \ge 0.86 )$ apparently reflects use of differing magnitudes of $\epsilon ,$ $\sigma ,$ and *M*,  in this article^41^ and in^38^. For example, depending on the technique used, empirical values of $\epsilon$ vary by approximately 7 %^41^ .

### Tangential electron cloud distortion and single- molecule-scale viscosity emergence

In liquid state physics, the single-molecule-scale mechanisms that trigger emergence of viscosity and viscous/frictional forces on individual molecules remains an open question. In this section and in the Supplement, focusing on nonpolar, nonmetallic liquids, we present evidence and arguments supporting the following hypotheses, each of which address this question.

- Under the nonrestrictive condition where nonequilibrium microscale momentum currents are produced by continuum scale velocity gradients, viscosity emerges on *single-molecule length scales.*
- Under the same conditions, viscosity appears due to sustained tangential distortion of interacting electron clouds.
- Viscosity emerges on time scales intermediate between the dispersion time scale, $\tau _d = O \left( 10^{-16} \mathrm {s} \right) ,$ and the Frenkel scale, $\tau _F = O \left( 10^{-14} \mathrm {s} \right) .$

Considering hypothesis (a), we first show (Supplement 1) that the Navier–Stokes equations, describing the collective, ensemble average dynamics of $N-\mathrm {molecule}$ (Newtonian) fluid systems can be adapted to single-molecule-scale (SMS) systems. This step provides the necessary theoretical framework for defining an SMS viscosity. Once this step is completed, we then show (Supplement 2)—here in preliminary fashion—that the Green–Kubo viscosity relation^22,33,35^, applied to *single-molecule-scale* (ensemble average) variations in the transverse momentum current leads to a parametrically correct relationship between the transverse momentum current correlation function, $\left\langle P_{xy} \left( 0 \right) P_{xy} \left( t \right) \right\rangle ,$ and the dynamic viscosity:

44 $$\begin{aligned} \int _0^{\infty } \left\langle P_{xy} \left( 0 \right) P_{xy} \left( t \right) \right\rangle dt \approx \frac{\mu ^2}{\delta y^2} \left\langle u_1^2 \left( \delta y ,0 \right) \right\rangle \int _0^{\infty } e^{- \gamma t'} dt' = \frac{\mu ^2}{\delta y^2} \frac{ u_0^2}{\gamma } = \frac{\mu }{3 \pi \sigma _o^3 c_o^2 \beta } \end{aligned}$$

where the meaning of all terms is given in Supplement 2. Together, these steps provide strong evidence that dynamic viscosity, at least in nonpolar liquids satisfying Newtonian constitutive relationships^33,71^, emerges, and can be defined on single molecule length scales.

**Figure 7:**
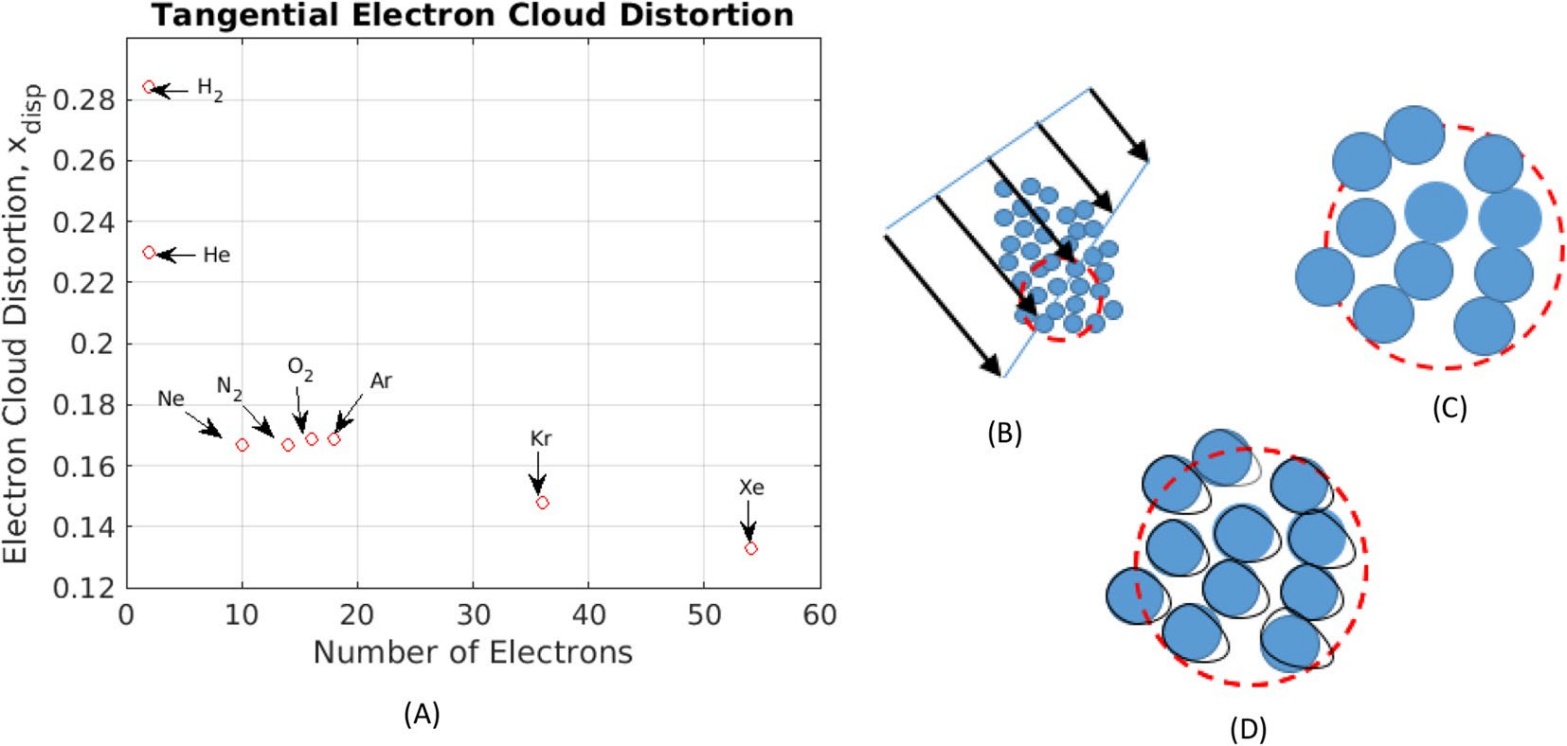
Distortion of electron clouds appears to play a dominant role in both emergence of single-molecule-scale viscosity and resistive viscous forces, as well as in repulsion-driven single molecule hopping. Intuitively, we anticipate that the magnitudes of cloud distortion associated with each process, $\delta \sigma _{disp}$ and $\delta \sigma _{diff} ,$ respectively, should be roughly of the same magnitude. In order to test this idea, and as a consistency check on the proposed models of dispersion-induced viscosity generation and phonon-induced self-diffusion, we estimate viscosity-generating cloud distortion, $\delta \sigma _{disp} ,$ using an expression equivalent to (8) for the polarization^41^: $\alpha = 4 n \langle r_1^2 \rangle ^2 / \left( 9 a_o \right) ,$ where *n* is the number of electrons in the molecule, and $\langle r_1^2 \rangle /$ is the mean squared collision-induced displacement of any of the (indistinguishable) electrons occupying the first shell of the molecule. Labeling the quartic root of the latter as $\delta \sigma _{disp} ,$ and identifying this as the characteristic *tangential* cloud distortion, we calculate the relative tangential distortion as: $x_{disp} = \delta \sigma _{disp} / \sigma .$ As shown in (**A**), outside of He and $\mathrm {H_2}$ - which, due to small masses, are apparently dominated by quantum collision dynamics - estimated tangential cloud distortions are approximately of the same magnitiude as those producing self-diffusion, Fig. 6. Thus, while cloud distortions are of comparable magnitude, the *type* of distortion, compressive versus shearing, engages repulsive versus attractive intermolecular forces. Plate (**B**) highlights the essential role of microscale (long-time-averaged) shear stresses in driving tangential cloud distortion and resulting viscosity generation. Plate (**C**) depicts liquid state molecules under local equilibrium conditions (sans phonon jitter). Nonequilibrium, shear-driven, tangential cloud distortion appears as small white areas at the edge of each molecule in Plate (**D**).

The argument supporting hypothesis (b), proceeds as follows. Again, in nonpolar, non-metallic liquids, London dispersion constitutes the *only* intermolecular force extant. While dispersion arises as a quantum mechanical perturbation to initially independent ground state wave functions^41,44^, as described above, London’s model can also be couched in terms of molecular polarizabilities, i.e., electron cloud distortions^41,44^. Importantly, this picture underlies the simple model used to derive theoretical viscosities, (14), above. Thus, in light of the estimates of electron cloud compression, $x_{diff,1}$ and $x_{diff,2}$ - which provide physically distinct, but self-consistent evidence of cloud-compression-induced self-diffusion—we hypothesize that *tangential* electron cloud distortion underlies the emergence of viscosity. A schematic representation of this hypothesis is shown in Fig. 7B–D.

As a rough quantitative test of this picture, we estimate relative electron cloud distortions, $$ x_{disp} , $$ induced by persistent nonequilibrium shear stress. Given that surrounding (bath) molecules exist in a state of near-equilibrium, at temperature *T* and mean kinetic energy, $$ 3 k_B T / 2 , $$ we anticipate that relative tangential cloud distortions should be of the same order of magnitude as normal distortions, $$ x_{diff,1} $$ and $$ x_{diff,2} ,$$ estimated above. Thus, combining (8) and (11), we arrive at:

45 $$\begin{aligned} x_{disp} = \frac{\delta \sigma }{\sigma } \approx \left[ \frac{9}{4} \frac{ \alpha a_o}{n} \right] ^{1/4} \sigma ^{-1} \end{aligned}$$

As shown in Fig. 7, estimated relative, shear-induced electron cloud distortion, for a number of noble and diatomic liquids, are approximately of the same magnitude as $$ x_{diff,1} $$ and $$ x_{diff,2} .$$ Dynamically, due to the cohesive nature of viscosity, shear-induced separation of interacting nuclear pairs, $$ r_{\mathscr {AB}}, $$ exceeds the potential-minimizing separation, $$ 2^{1/6} \sigma . $$ Finally, note that use of the definition of $$ \delta \sigma ,$$ given by (11), means that we are approximating the set of level-dependent mean squared electron displacements, $$ \overline{r_i^2} , $$ as $$ \overline{r_1^2} $$^41^; thus, plotted magnitudes of $$ x_{disp} $$ represent slight underestimates.

The argument supporting hypothesis c) is presented as Note b) in the final section. We note that derivation^71^ of the single-molecule-scale Stokes (viscous) drag force, *D*,  that appears in the modified Stoke’s–Einstein relation^46^, (4), requires use of the SMS Navier-Stokes equations derived in Supplement 1.

In closing this section, we remark that while computational techniques^66,68,72,73^ can be used to test the proposed connections between normal and tangential electron cloud distortion and self-diffusion and viscosity emergence, we argue that simple, approximate models like those proposed here, are valuable in providing physical insight into the mechanisms that drive self-diffusion and viscosity emergence.

## Time scale-dependent models of single molecule dynamics

For nonpolar, spherical atomic liquids like Ar, Kr, and Xe, as well as nonspherical, nonpolar liquids like those examined in^46^, we propose that single molecule dynamics can be modeled on three distinct time scales: (a) over $\tau _d \lesssim t \lesssim \tau _F ,$ dynamics are solid-state-like and either dissipative or not—see below; (b) over $\tau _F \lesssim t \lesssim \tau _c ,$ dynamics are a mixture of solid- and fluid-like and again, either dissipative or not; and c) for $t > \tau _c ,$ dynamics are dissipative and fluid-like. We denote these time scales, respectively, as the solid-like, transitional, and fluid-like regimes.

For clarity, we tabulate in Table 2 the various forms that the single molecule dynamics equation can take. The following general points are highlighted:

**(a) Equation structure:** The proposed equations are physically explicit versions of the memory-free and generalized Langevin equation^22,33,35^:

46 $$\begin{aligned} M \frac{ d {\mathbf {v}} \left( t \right) }{dt} = \mathbf {F_e} \left( t \right) + \mathbf {F_f} \left( t \right) + \mathbf {F_R} \left( t \right) \end{aligned}$$

where the instantaneous molecular force is decomposed into a time-dependent external force, $\mathbf {F_e} \left( t \right) ,$ necessary for modeling, e.g., particle scattering problems, a deterministic friction force, $\mathbf {F_f} \left( t \right) ,$ either $- \int _0^t \kappa \left( t - t' \right) {\mathbf {v}} \left( t' \right) dt'$ or $- 3 \pi \sigma _m \mu {\mathbf {v}} \left( t \right) ,$ and a random force, $\mathbf {F_R} \left( t \right) ,$ either determined by the phonon field, $M \sum _{i=1} \omega _i^2 \int _0^t \tilde{{\mathbf {v}}}_i \left( t , \tau \right) d \tau ,$ or by the thermal motion of surrounding molecules, $\dot{\varvec{\eta }} \left( t \right) .$

**(b) Friction force:** The set of equations contrasts the qualitatively distinct dynamics that exist under quiescent conditions, when the continuum-scale liquid flows or is stationary, versus the highly dynamic state extant when the target molecule interacts directly with, or lies near an externally introduced particle. In the first case, based on the observation that small molecules follow a slightly modified version of the Stokes-Einstein drag force law^46^, we assume that the friction force can be expressed as $- 3 \pi \sigma _m \mu {\mathbf {v}} \left( t \right) ,$ where again, $\sigma _m$ is an effective, shape-dependent molecular diameter. This assumption, in turn, assumes that the dynamic viscosity, $\mu ,$ emerges on time scales that are long relative to the fast disperion time scale, $\tau _d ,$ but short relative to the solid-liquid cross-over time scale, $\tau _F .$ Based on the observation that temperature-dependent viscosities are well-predicted by the above model incorporating fast-acting dispersion forces, this appears to be a reasonable assumption. Under conditions where, e.g., scattering particles interact with or near the target molecule, numerous experimental observations show that the friction force is history dependent^20–22,33^.

**(c) Connecting the phonon-induced force to the Brownian force:** Kubo’s analysis^35^ can be adapted to show explicitly how the phonon-induced force on $\tau _d \lesssim t \lesssim \tau _F$ can be represented as a Brownian force on $t \gtrsim \tau _c :$ i) Express the instantaneous phonon-induced force (in any of three orthogonal directions) as $F_{phonon} \left( t \right) = M \sum _{i=1}^n \omega _i^2 \Delta x_i ,$ where $\Delta x_i \left( t \right) = \int _0^t \tilde{{\mathbf {v}}}_i \left( t , \tau \right) d \tau ,$ is the nuclear displacement produced by phonon mode *i*,  and $\omega _i$ the $\mathrm {i^{th}}$ normal mode frequency; ii) recognize, by (normal mode) construction, that on $\tau _d \lesssim t \lesssim \tau _F ,$ all $\Delta x_i ' \mathrm {s}$ are zero-mean, independent random displacements; iii) define the sum of displacement variances as $s_n^2 = \sum _{i=1}^n \sigma _i^2 ,$ where $\sigma _i^2 = \langle \Delta x_i^2 \rangle$ is the $\mathrm {i^{th}}$ variance; iv) focusing on time scales on the order of $\tau _c$ and longer, define a random variable $Y_n \left( t \right) = F_{phonon} \left( t \right) / s_n ,$ where $t = \mathrm {O} \left( \tau _c \right) ;$ v) allow *n* to become large, which corresponds to binning all of the random phonon-induced forces acting on $\tau _d \lesssim t \lesssim \tau _F ;$ vi) by the central limit theorem, the probability density for $Y_n \left( t \right)$ approaches a Gaussian density, $p \left( Y \left( t \right) \right) \rightarrow \frac{1}{\sqrt{2 \pi } } \exp { \frac{ -Y^2 }{2}} ;$ and vii) on $t = \mathrm {O} \left( \tau _c \right) ,$ argue that phonon-induced force components (in each of three orthogonal directions) are delta-correlated, $\langle F_{phonon} \left( t \right) F_{phonon} \left( t' \right) \rangle = F_o^2 \delta \left( t - t' \right) ,$ where $F_o^2$ is the force intensity. In Table 2, this guassian, delta-correlated remnant of the phonon-induced force is labeled as ${\dot{\varvec{\eta }}} \left( t \right) .$

**(d) The random force,** $\mathbf {F_R} \left( t \right) :$ For the solid-like regime, the arguments from the previous section provide, we believe, substantial support for expressing the random force as $\mathbf {F_R} \left( t \right) = -M \sum _{i=1} \omega _i^2 \int _0^t \tilde{{\mathbf {v}}}_i \left( t , \tau \right) d \tau .$ For the fluid-like regime, the fact that the modified Stokes-Einstein relation, (4) holds for a large family of molecules^46^, where again (4) is derivable from the memory-free Langevin equation (5)^47^, suggests that $\mathbf {F_R} \left( t \right) = {\dot{\varvec{\eta }}} \left( t \right) .$ Proposing a reasonable form of $\mathbf {F_R} \left( t \right)$ over the transition regime, $\tau _F \lesssim t \lesssim \tau _c ,$ remains problematic at this point, however. A mathematically simple assumption, which may not be physically valid, would model $\mathbf {F_R} \left( t \right)$ as a linear superposition of $-\sum _{i=1} \omega _i^2 \int _0^t \tilde{{\mathbf {v}}}_i \left( t , \tau \right) d \tau$ and ${\dot{\varvec{\eta }}} \left( t \right) .$ This is an open question, however.

**(e) The external force:** An external force term only appears for problems in which the spatial scale of the external agent, e.g., a scattering particle or a high-energy photon source (having wavelength on the order of $\sigma$ or smaller), is on the order of the molecular diameter, $\sigma .$ To account for such forces, a quantum mechanical model of the interaction is typically required; see, e.g.,^20,21^.

**(f) Physical meaning of the phonon-induced force:** Over the solid-state-like time scale, $\tau _d \lesssim t \lesssim \tau _F ,$ under conditions where molecule-scale external forcing is absent, the phonon field determines: (i) each molecule’s instantaneous velocity, ${\mathbf {v}} \left( t \right) = \sum _{i} {\tilde{\mathbf {v}}_j} \left( t, \omega _j \right) ,$ as well as ii) the instantaneous random force, $\mathbf {F_R} \left( t \right) = - M \sum _j \omega _j^2 \Delta {\tilde{\mathbf {x}}_j} \left( t \right) = - M \sum _j \omega _j^2 \int _0^t {\tilde{\mathbf {v}}_j} \left( t', \omega _j \right) dt' .$ Thus, the dynamics of individual nuclei: i) can be decomposed into individual contributions produced by each phonon mode: $M \dot{{\tilde{\mathbf {v}}}_j} = - M \omega _j^2 \int _0^t {\tilde{\mathbf {v}}_j} \left( t', \omega _j \right) dt' ,$ or ii) taken as the resultant of these modes: $M \dot{{\mathbf {v}}} = - M \sum _j \omega _j^2 \int _0^t {\tilde{\mathbf {v}}_j} \left( t', \omega _j \right) dt' ,$ where time derivatives, denoted by dots, are taken with respect *t*,  on the solid state time scale.

**(g) On the weak coupling between continuum scale flow and microscale dynamics:** A scaling argument shows that only under extreme circumstances can continuum flow fields produce non-negligible microscale nonequilibrium mass, momentum and energy currents. Consider, for example, turbulent flow over a mirror-smooth surface (having asperities on the order of, say, $10^{-9} \ \mathrm {m} ).$ Taking the ratio of the maximum continuum-scale viscous shear stress, evaluated at the surface, $\tau _{cont} \approx 0.02 \rho U_{\infty }^2 Re_{\delta }^{-1/4}$^74^, to the characteristic molecular-scale shear stress, $\tau _{molec} \approx 10 \mu a / \sigma ,$ leads to $\tau _{cont} / \tau _{molec} \sim 0.002 U_{\infty } Ma \sigma ,$ where $Re_{\delta } = \rho U_{\infty } \delta / \mu \sim 1$ is the Reynolds number associated with a turbulent boundary layer of thickness, $\delta ,$ $U_{\infty }$ is the speed of the flow external to the boundary layer, and $Ma = U_{\infty } / a ,$ is the associated Mach number. Here, $\tau _{molec} ,$ which is determined by the transverse momentum current^22,35^, is most easily estimated using the Stoke’s drag law, $F_{drag} \approx 3 \pi \sigma \mu a$^71^, where the molecular speed is approximated as the sound speed, *a*. Using the Mach number magnitude, $Ma \sim 0.3 ,$ separating nominally incompressible and compressible flow, leads to the condition: $\tau _{cont} \sim \tau _{molec}$ when $U_{\infty } \sigma / nu \sim 10^3 .$ Due the small magnitude of $\sigma$ for small molecular species, it is found, for Ar, Kr, and Xe, that $U_{\infty }$ must be on the order of $10^6 \ \mathrm {m/s} ,$ or higher for continuum-scale dynamics to manifest itself in microscale dynamics.

**(h) Development of short time scale collective dynamics models:** Under the assumption that fast-acting dispersion forces mediate collective dynamics over the elastic, transition, and fluid-like regimes, sum rules^20,22,33,35^ provide a powerful tool for developing hydrodynamic models appropriate to each time scale. Supplement 5 illustrates using a simplified, i.e., non-viscoelastic Navier Stokes model of transition regime collective dynamics. The strategy consists of two steps: (1) propose a model of short-time scale (ensemble average) molecular hydrodynamics, and (2) constrain the model by satisfying sum rules.

**(i) Dominance of pairwise interactions:** In many single molecule dynamics problems, as well as in derivation of field-based continuum dynamics models^31,33^, it is important to have solid understanding of the relative importance of simultaneous multi-molecule collisions. At any instant, on any time scale exceeding $$ \tau _d , $$ consider a target (nonpolar, liquid-state) molecule, $$ \mathscr {A,} $$ surrounded by a set of neighboring molecules, $$ {\mathscr {B}}_1 , {\mathscr {B}}_2 ,\ldots , {\mathscr {B}}_m . $$ Since the weak dispersive potential, $$ \phi ^{(AB_i)} , $$ that appears during collision of $$ {\mathscr {A}} $$ and $$ {\mathscr {B}}_i , $$ is small relative to the ground state energy, $$ E_A^{(0)} + E_{B_i}^{(0)} , $$ of adjacent, but unperturbed $$ {\mathscr {A}} $$ and $$ {\mathscr {B}}_i , $$ the London collision model^41,44^ is linear and can be readily modified by superposition to account for n-body interactions in which $$ {\mathscr {A}} $$ simultaneously experiences dispersive interactions with *n* neighboring molecules. Generalizing Hirschfelder^41^ by assuming a perturbed wave function that is the product of the *n* unperturbed, isolated wave functions for *n* colliding molecules, it is readily shown that the approximate, second-order, dispersive potential has the form,

47 $$\begin{aligned}&\phi ^{(n)} \left( \mathbf {r_A} , \mathbf {r_2} ,\ldots , \mathbf {r_{n-1}} \right) = \nonumber \\&\quad -\left( 3/2 \right) E_I \alpha ^2 \left[ \mathbf {r_{A1}}^{-6} + \mathbf {r_{A2}}^{-6} +\cdots + \mathbf {r_{Am}}^{-6} \right] \end{aligned}$$

where $$ E_I , $$ an empirical constant, is on the order of the ionization energy, $$ \alpha $$ is the polarizability, $$ \mathbf {r_{Ai}} $$ is the internuclear distance between molecules $$ {\mathscr {A}} $$ and $$ {\mathscr {B}}_i , $$ and $$ m=n-1 .$$

A ’simultaneous n-body collision’ takes place when the internuclear distances between ${\mathscr {A}}$ and $n-1$ immediately adjacent molecules are all approximately equal to the minimum of these distances, $\mathbf {r_{A1}} \approx \mathbf {r_{A2}} \approx ... \approx \mathbf {r_{Ai_{min}}}.$ Writing $\mathbf {r_{Aj}} = \mathbf {r_{Ai_{min}}} + \Delta \mathbf {r_{Aj}} ,$ forming the ratio $\mathbf {r_{Aj}} / \mathbf {r_{Ai_{min}}} ,$ and Taylor expanding $\mathbf {r_{Aj}} ,$ we see that for an n-body collision to occur - corresponding to *n* non-negligible contributions to $\phi ^{(n)} \left( \mathbf {r_A} , \mathbf {r_2} ,\ldots , \mathbf {r_{n-1}} \right)$- all $n-1$ molecules must remain within approximately $16 \%$ of the minimum separation, $\mathbf {r_{Ai_{min}}}.$ Thus, while three-body collisions certainly take place, for example, due to this restrictive condition, pair-wise collisions dominate. Predicted dynamic viscosities above, which assume dominant pairwise collisions, are consistent with this simple argument.

**Table 2:** Situation- and time-scale-dependent force terms can be inserted into the generic Langevin equation, (46).

Single molecule dynamics models				
Time scale [Collective dynamics]	Dominant physics [Example]	External force	Friction force (deterministic)	Random force
$\tau _d \lesssim t \lesssim \tau _F$	Phonons; low dissipation			
[Elastic]	[Continuum flow]	N/A	$- 3 \pi \sigma _m \mu {\mathbf {v}} \left( t \right) \rightarrow 0$	$-M \sum _{i=1} \omega _i^2 \int _{0}^t \tilde{{\mathbf {v}}}_i \left( t , \tau \right) d \tau$
$\tau _d \lesssim t \lesssim \tau _F$	Phonons; dissipation			
[Viscoelastic]	[Particle scattering]	${\mathbf {F}} \left( t \right)$	$- \int _0^t \kappa \left( t - t' \right) {\mathbf {v}} \left( t' \right) dt'$	$- M \sum _{i=1} \omega _i^2 \int _{0}^t \tilde{{\mathbf {v}}}_i \left( t , \tau \right) d \tau$
$\tau _F \lesssim t \lesssim \tau _c$	Phonons; low dissipation			
[Transition]	[Continuum flow]	N/A	$- 3 \pi \sigma _m \mu {\mathbf {v}} \left( t \right) \rightarrow 0$	See remark d)
$\tau _F \lesssim t \lesssim \tau _c$	Phonons; dissipation			
[Viscoelastic]	[Particle scattering]	${\mathbf {F}} \left( t \right)$	$- \int _{0}^t \kappa \left( t - t' \right) {\mathbf {v}} \left( t' \right) dt'$	See remark d)
$t \gtrsim \tau _c$	Brownian force; dissipation			
[Fluid]	[Continuum flow]	N/A	$- 3 \pi \sigma _m \mu {\mathbf {v}} \left( t \right)$	${\dot{\eta }} \left( t \right)$
$t \gtrsim \tau _c$	Brownian force; dissipation			
[Viscoelastic]	[Particle scattering]	${\mathbf {F}} \left( t \right)$	$- \int _0^t \kappa \left( t - t' \right) {\mathbf {v}} \left( t' \right) dt'$	${\dot{\eta }} \left( t \right)$

Explanatory notes regarding each force term are given above as points (a) through (i).

## Conclusions

Unraveling the dynamics of individual atoms and small molecules in liquids represents a centuries-old physics problem. While neutron and light-scattering experiments, as well as molecular dynamics simulations, instantaneous normal mode analyses, and molecular hydrodynamics expose and explain single-particle-scale and collective liquid-state dynamics, the descriptions are largely couched in terms of dynamical correlation functions. In an attempt to expose the essential dynamical elements that determine single molecule motion, at least in nonpolar, nonmetallic liquids, this paper presents physical arguments that suggest: (i) intermolecular dispersion forces and temperature-dependent electron screening determine viscosity, i.e., temperature-dependent intermolecular friction forces, and (ii) a narrow band of phonons, lying near the liquid-solid (Frenkel) transition frequency, drives the random molecular jumps constituting self-diffusion.

In mechanistic terms, we present preliminary evidence that, in simple liquids, both viscosity and single molecule viscous drag emerge due to small, collision-induced *tangential* distortions of individual electron clouds. By contrast, self-diffusional, single-molecule hops are produced by collision-induced *compression* of interacting molecular clouds; the latter mechanism pushes interacting molecular pairs into short-lived repulsive energy states.

We are hopeful that the preliminary picture of single molecule, liquid state dynamics proposed here promotes further progress in understanding this complex problem.

## Supplementary Information

Supplementary information.
