# Supplementary material for: On the physical mechanisms underlying single molecule dynamics in simple liquids
Source: Sci Rep. 2021 Jan 28;11:2528. doi: 10.1038/s41598-021-82112-8 (PMC7843658; doi:10.1038/s41598-021-82112-8)
Supplement: Supplementary file 1 — Supplementary information. [file 41598_2021_82112_MOESM1_ESM.pdf]

Supplemental Information - On the physical mechanisms underlying single molecule dynamics in simple liquids

Russell G. Keanini[1], Jerry Dahlberg[1], and Peter T. Tkacik[1]

[1]University of North Carolina at Charlotte, Department of Mechanical Engineering, Charlotte, 28078, United States

Supplement 1: Derivation of single-molecule-scale Navier-Stokes equations

Overview

In the following, as in the article, characteristic time scales are those associated with liquid Ar, Kr and Xe. The model we propose derives from four observations:

a) In the presence of surrounding liquid state molecules, electron distributions of individual molecules undergo extremely rapid, dispersion-time-scale, $\tau_d = O\left(10^{-16}\text{ s}\right)$, distortion. This indicates that single-molecule-scale (SMS) equilibrium exists on time scales of order $\sim 10\tau_d$ and longer.

b) As argued in Supplement 3, viscosity and viscous forces emerge on time-scales, $\tau_I = O\left(10^{-15}\text{ s}\right)$, much shorter than the intermolecular collision time-scale, $\tau_c = O\left(10^{-13}\text{ s}\right)$.

c) A modified Stokes-Einstein relation, given in the article, which accounts for molecular shape[1], is found to hold for a large family of single molecules. Since the (viscous) drag force that appears in this relationship corresponds to the drag on a single molecule, and since the drag can be derived from the Navier-Stokes equations[2], this observation suggests that the Navier-Stokes equations apply on single-molecule length scales. Additionally, in light of b), the equations apply on subcollision time-scales, $\tau_d << t \lesssim \tau_c$.

d) As shown in[3,4], equilibrium and nonequilibrium statistical mechanical arguments can be adapted to derive field-based Navier-Stokes equations for predicting the collective, long-time-scale, flow-like dynamics observed in vibrated grain beds[5]. Since these systems possess many of the known collective and single-particle scale dynamics observed and predicted in dense molecular hydrodynamic systems[3], they represent macroscopic dynamical analogs of the latter[3,4]. Thus, the observation that long-time-averaged, single-grain-scale dynamics are well-predicted by grain system NS equations[3,5] leads us to believe that similar results hold between ensemble averaged, SMS (field-based) dynamics and SMS Navier-Stokes equations.

In this Supplement, we employ the same arguments used in[3] to develop Navier-stokes equations describing the flow of vibration-driven grain beds. It is important to recognize that, in contrast to the particle based Langevin models proposed in the article, the SMS Navier-Stokes equations developed here provide a *field-based,* ensemble averaged description of fluid dynamics on single molecule length scales.

In order to extend the Navier-Stokes equations to the ensemble average motion of single-molecule-scale, liquid-state systems, several steps are required:

1) First, basic definitions and concepts must be stated. These include definitions of single-particle mass and momentum densities and currents, as well as ensemble averages of these quantities.

2) Second, the exact equations describing the linear response of a single, liquid-state molecule to a local perturbation, must be derived. The response equations, which incorporate the dynamics of the molecules surrounding the target molecule, correspond to the mass and momentum conservation equations, and when solved, yield the ensemble-averaged mass and momentum densities of the target molecule, each stated as a time-varying field variable.

3) Third, derivation of the Navier-Stokes equations from the linear response equations requires a central assumption that the single-molecule system always exists at or near a state of local statistical mechanical and thermodynamic equilibrium. This essential attribute allows recasting of unobservable terms in molecular-scale mass and momentum densities and currents in terms of equilibrium thermodynamic properties, specifically, density and pressure.

4) Once steps 1) through 3) are complete, and based on observations in vibrated grain beds[3,4], we assume that Newtonian constitutive relationships hold on single molecule scales. On substitution into the conservation equations in step 2), we obtain the SMS Navier-Stokes equations, which govern the evolution of the ensemble average, single-molecule-scale mass and momentum densities; again, these are applicable on time-scales on the order of the intermediate-time-scale, τ_I, and longer.

Note: In this Supplement, we do not consider derivation of the single-molecule-scale energy equation. This will be presented in another publication.

Definitions and concepts

Consider the sub-collision-time-scale, ensemble averaged motion of a single liquid-state molecule, *relative* to the ensemble (or time-) average motion an encompassing, multi-molecule *fluid particle*. The single-molecule-scale Navier-Stokes equations derived in this Supplement predict the field-based linear response, i.e., the space- and time-dependent, ensemble average, single-molecule-scale evolution of the mass and momentum density fields following an arbitrary perturbation from single-molecule-scale equilibrium.

a) We define a *fluid particle* (FP) as a fixed mass collection of N adjacent molecules. For concreteness, at any instant, t, imagine a cubical collection of say, $N = O\left(10^3\right)$ spherical molecules, each of approximate diameter, $\sigma_o \approx 3\left(10^{-10}\right)$ m. Letting $l_p \approx N^{1/3}/\sigma_o$, be the fluid particle's approximate length and D_s the characteristic self-diffusion coefficient, then on time-scales shorter than $\tau_{FP} = \sigma_o^2/D_s \lesssim 10^{-10}$ s, the molecules comprising the fluid particle experience limited thermal diffusion and the FP remains coherent and non-dispersed, where $D_s \approx 2\left(10^{-10}\ \mathrm{m^2 s^{-1}}\right)$ for, e.g., liquid Ar[6]. In the following, we focus on the dynamics of an arbitrary single molecule situated within the FP, taking place on intermediate and longer time-scales, $\tau_I = O\left(10^{-15}\right) << \tau_{FP}$.

b) In a slight abuse of convention, we will refer to the ensemble average velocity of single molecules, relative to that of the encompassing fluid particle, as the *peculiar velocity*.

c) We will often refer to *single-molecule* mass and momentum densities and currents. The term *single-molecule* or *single-molecule-scale* refers to any variable or property that varies on single-molecule length scales. The ensemble average space-time variation of these variables and properties determines associated variable and property fields.

Mass and momentum densities

We follow Forster[7], adapting his definitions of number and momentum density for $N-$particle systems to single-molecule systems. At any instant, t, at an arbitrary location, $\mathbf{r_{fp}}$, within a given (turbulent or laminar) flow, define a small FP, containing N molecules. Subdivide the FP into N equal subvolumes, $\delta V_i,\ i = 1,...N$, where δV_i is approximately equal to the nominal volume of a single molecule, and label the location of each subvolume's centroid as $\mathrm{r_i},\ i = 1,...,N$.

Now imagine an ensemble of $\mathscr{N}$ similar FP's, each containing N molecules, each again subdivided into N subvolumes, where the set of subvolume centroid locations, $(\mathbf{r_1},...,\mathbf{r_N})_\kappa,\ \kappa = 1,...,\mathscr{N}$, is fixed for all member FP's in the ensemble: $\mathbf{r_i}^\kappa = \mathbf{r_i}^\lambda,\ i = 1,...,N;\ \kappa,\lambda = 1,...,\mathscr{N}, \kappa \neq \lambda$.

The respective single-molecule number and momentum densities are thus expressed as:

$$n(\mathbf{r_i},t) = \sum_{\kappa}^{\mathscr{N}} \delta\left(\mathbf{r_i} - \mathbf{r}^\kappa(t)\right) \tag{1}$$

$$\mathbf{j}(\mathbf{r_i},t) = \sum_{\kappa=1}^{\mathscr{N}} M\mathbf{v}^\kappa(t)\,\delta\left(\mathbf{r_i} - \mathbf{r}^\kappa(t)\right) \tag{2}$$

Here, the function $\delta\left(\mathbf{r_i} - \mathbf{r}^\kappa(t)\right)$ performs two tasks: i) In the κth composite system, FP^κ, of the ensemble, the function picks out the single molecule, $\mathscr{M}_i^\kappa$, having center of mass nearest $\mathbf{r_i}$, and ii) assigns a volume fraction, $\phi_\kappa(\mathbf{r_i})$ - defined as the

temperature- (and weakly pressure-) dependent volume of $\mathscr{M}_i^\kappa$ located within subvolume δV_i - to δV_i. As argued in the article, in the liquid state, for nonpolar molecules, the nominal diameter of a molecule's electron distribution decreases approximately linearly with temperature, T, indicating that molecular volume decreases approximately as T^3. The weak dependence of molecular volume on pressure can be seen in the corresponding states correlation[8].

In addition, $\mathbf{P}^\kappa$, M, $r_{\kappa q} = \left|\mathbf{r_q}^\kappa - \mathbf{r}^\kappa\right|$, and $V\left(r_{\kappa q}\right)$, represent, respectively, the momentum and mass of molecule $\mathscr{M}_i^\kappa$, the distance between the chosen molecule, $\mathscr{M}_i^\kappa$, nearest $\mathbf{r_i}$ in FP$^\kappa$, and the qth molecule, also in FP$^\kappa$, and the associated intermolecular potential. Note that the sum of potentials within FP$^\kappa$ doesn't include the usual leading coefficient, $1/2$, and, likewise, doesn't include molecule $\mathscr{M}_i^\kappa$ in the sum. Finally, the instantaneous position and velocity of $\mathscr{M}_i^\kappa$ are related by $\mathbf{v}^\kappa(t) = d\mathbf{r}^\kappa/dt\,(t)$.

Given (1) and (2), associated ensemble averaged single-molecule number and momentum densities, determined at time t and point $\mathbf{r_i}$, are given by:

$$\langle n(\mathbf{r_i},t)\rangle = \frac{1}{\mathscr{N}} n(\mathbf{r_i},t) \tag{3}$$

$$\langle \mathbf{j}(\mathbf{r_i},t)\rangle = \frac{1}{\mathscr{N}} \mathbf{J}(\mathbf{r_i},t) \tag{4}$$

The ensemble averaged single-molecule number density thus represents the average molecular volume occupying subvolume δV_i. Similarly, the averaged single-molecule-scale momentum density represents the average momentum of the ensemble of $\mathscr{N}$ individual molecules located nearest $\mathbf{r_i}$.

Single-molecule-scale mass conservation equation

In deriving the single-molecule-scale, field-based conservation laws, in this section we use an approach given by[9] to derive the SMS mass conservation equation. In deriving the single-molecule-scale momentum equation below, we use an alternative, continuum-mechanics-, i.e., field-based argument that circumvents consideration[9] of spatial derivatives of the sifting function, $\delta\left(\mathbf{r_i} - \mathbf{r}^\kappa(t)\right)$.

Define the local, ensemble average, single-molecule-scale mass density as

$$\rho(\mathbf{r_i},t) = M\langle n(\mathbf{r_i},t)\rangle = \frac{1}{\mathscr{N}}\sum_{\kappa}^{\mathscr{N}} M\delta\left(\mathbf{r_i} - \mathbf{r}^\kappa(t)\right) \tag{5}$$

where again $\delta\left(\mathbf{r_i} - \mathbf{r}^\kappa(t)\right)$ has the sifting property described above: $\mathbf{r}^\kappa = \mathbf{r}^\kappa(t)$ is the instantaneous position of the molecule - in the κth fluid particle of the ensemble - having center of mass nearest $\mathbf{r}$.

Next take the spatial Fourier transform of (5), but first shrink the subvolumes δV_i to dimensions much smaller than the characteristic molecular diameter, σ_o. This step is required to ensure that the Fourier transform, which effectively sums instantaneous dynamics extant over many subvolumes, only sums dynamics on single-molecule length scales. The step is rigorously justified when we recognize that under nonequilibrium conditions, fluid-particle-scale, ensemble average spatial property variations imply sub-molecular-length-scale variations in the same properties. Mathematically, and on length scales larger than a few molecular diameters, shrinking spatial dimensions produces rapidly varying sine terms, that on summation, cancel in Fourier transforms. We thus obtain:

$$\tilde{\rho}(\mathbf{k},t) = \frac{1}{\mathscr{N}}\int_{-\infty}^{\infty} \exp(i\mathbf{k}\cdot\mathbf{r})\sum_{\kappa=1}^{\mathscr{N}} M\delta\left(\mathbf{r} - \mathbf{r}^\kappa(t)\right)d\mathbf{r} = \frac{1}{\mathscr{N}}\sum_{\kappa=1}^{\mathscr{N}} M\exp\left(i\mathbf{k}\cdot\mathbf{r}^\kappa\right) \tag{6}$$

Note: The definition for $\rho(\mathbf{r},t)$ comprises a *constitutive relation*, an assumed connection between a potentially observable (molecular-scale) property, $\rho(\mathbf{r},t)$, and a set of unobservable molecular-scale properties, i.e., the set of individual molecular positions within any given FP.

Next, introduce a second constitutive relation by defining the ensemble average, single-molecule velocity, $\mathbf{u}(\mathbf{r},t)$:

$$\rho(\mathbf{r},t)\,\mathbf{u}(\mathbf{r},t) = \langle \mathbf{j}(\mathbf{r},t)\rangle \tag{7}$$

where $\langle\mathbf{j}\rangle$ is given by (4). Rigorously, $\mathbf{u}(\mathbf{r},t)$ corresponds to the ensemble average momentum density of all the individual molecules nearest $\mathbf{r}$ at t, normalized by the associated mass density. Approximately, $\mathbf{u}(\mathbf{r},t)$ represents the ensemble average

velocity of these molecules. Taking the Fourier transform then yields

$$\int_{-\infty}^{\infty} \exp(i\mathbf{k}\cdot\mathbf{r})\rho(\mathbf{r},t)\,\mathbf{u}(\mathbf{r},t)\,\mathbf{dr} = \left\langle \tilde{\mathbf{j}}(\mathbf{k},t)\right\rangle = \frac{1}{\mathcal{N}}\sum_{\kappa=1}^{\mathcal{N}} \exp(i\mathbf{k}\cdot\mathbf{r}^{\kappa})m\mathbf{v}^{\kappa}(t) \tag{8}$$

From (6), the time derivative of $\tilde{\rho}(\mathbf{k},t)$,

$$\frac{\partial}{\partial t}\tilde{\rho}(\mathbf{k},t) = i\mathbf{k}\cdot\frac{1}{\mathcal{N}}\sum_{\kappa=1}^{\mathcal{N}} \exp(i\mathbf{k}\cdot\mathbf{r}^{\kappa})m\mathbf{v}^{\kappa}$$

or

$$\frac{\partial}{\partial t}\tilde{\rho}(\mathbf{k},t) = i\mathbf{k}\cdot\left\langle \tilde{\mathbf{j}}(\mathbf{k},t)\right\rangle \tag{9}$$

Taking the Fourier transform of the molecule-scale divergence of the momentum density, $\langle \mathbf{j}(\mathbf{r},t)\rangle$, we obtain

$$\begin{aligned}\int_{-\infty}^{\infty} \exp(i\mathbf{k}\cdot\mathbf{r})\nabla\cdot\mathbf{J}d\mathbf{r} &= \int_{-\infty}^{\infty} \left[\nabla\cdot(\exp(i\mathbf{k}\cdot\mathbf{r})\langle\mathbf{j}\rangle) - \langle\mathbf{j}\rangle\cdot\nabla\exp(i\mathbf{k}\cdot\mathbf{r})\right]d\mathbf{r}\\ &= \exp(i\mathbf{k}\cdot\mathbf{r})\langle\mathbf{j}\rangle S_{\infty} - \int_{-\infty}^{\infty}\langle\mathbf{j}\rangle\cdot\nabla\exp(i\mathbf{k}\cdot\mathbf{r})d\mathbf{r}\\ &= -i\mathbf{k}\cdot\int_{-\infty}^{\infty}\exp(i\mathbf{k}\cdot\mathbf{r})\langle\mathbf{j}\rangle d\mathbf{r}\\ &= -i\mathbf{k}\cdot\left\langle\tilde{\mathbf{j}}(\mathbf{k},t)\right\rangle\end{aligned} \tag{10}$$

In the second line, S_{∞} is the surface of a sphere having radius large relative to the characteristic molecular size, σ_o; in order to drop the first term on the right side of line 2, we must thus assume that the ensemble average, single-molecule-scale momentum flux, $\langle\mathbf{j}\rangle$, decays to evanescence on S_{∞}. This assumption means that we are modeling the response - of both the single molecule system and closely neighboring molecules - to small (linear) disturbances and fluctuations, again relative to the bulk motion of the encompassing FP. With regard to modeling single-molecule-scale linear response, this is not a limiting assumption; the response to any prescribed single-molecule-scale disturbance can be determined by superposition.

Finally, comparing the last line of (10) with (9), transforming back to physical space, we obtain the field-based, single-molecule-scale law of mass conservation:

$$\frac{\partial\rho}{\partial t} + \nabla\cdot\langle\mathbf{j}\rangle = 0 \tag{11}$$

Crucially, this argument, and a similar one applied to the ensemble average momentum density in the next section, demonstrates the following features:

a) Recasting traditional mass and momentum density definitions for N-particle systems as ensemble average, single-molecule-scale densities, and introducing traditional linear constitutive relations, leads to physically reasonable, mathematically consistent single-molecule-scale, field-based conservation laws, the latter being identical in form to the well-known N-particle versions.

b) The set of single-molecule-scale conservation laws, four in number, identical in form to the traditional N-particle Navier-Stokes equations, and governing the single-molecule-scale, linear evolution of the ensemble averaged mass and momentum density fields, can be used to model any molecule-scale dynamical problem and can be solved by any of the powerful techniques developed for tackling the linear, viscous Navier-Stokes equations[2].

c) The argument above and that below make clear that solutions for the mass density field, $\rho(\mathbf{r},t)$, as well as those for the velocity field, $\mathbf{u}(\mathbf{r},t)$, and pressure field, $P(\mathbf{r},t)$, represent the ensemble average, single-molecule-scale nonequilibrium response, taken over the set of single molecules nearest $\mathbf{r}$, at t.

Derivation of the single-molecule-scale, field-based momentum conservation equations

We begin by deriving a first version of the single-molecule-scale momentum equation, stated in terms of the ensemble-averaged intermolecular forces that produce single-molecule-scale, nonequilibrium dynamics. See (14) below. We then use a continuum-mechanics-based argument to arrive at a second version, (21). Comparison of (14) with (21) then leads to a useful identity, (23), connecting the field-based, i.e., coarse-grained force description in (21) to the detailed description in (14).

Thus, define the SMS ensemble average momentum density as

$$[\rho \mathbf{v}](\mathbf{r},t) = \left\langle \sum_{\kappa=1}^{\mathscr{N}} M\mathbf{v}^{\kappa}(t)\,\delta\left(\mathbf{r}-\mathbf{r}^{\kappa}(t)\right)\right\rangle \tag{12}$$

where M and $\mathbf{v}^{\kappa}(t)$ again represent the mass and velocity of the molecule nearest $\mathbf{r}$, at time t, in the κth member of the ensemble.

Taking the time-derivative of both sides of (12) then yields:

$$(\rho \mathbf{v})_t = \left\langle \sum_{\kappa=1}^{\mathscr{N}} M\dot{\mathbf{v}}^{\kappa}(t)\,\delta\left(\mathbf{r}-\mathbf{r}^{\kappa}(t)\right)\right\rangle - \left\langle \sum_{k=1}^{N} M\mathbf{v}^{\kappa}\left(\mathbf{v}^{\kappa}\cdot\nabla\right)\delta\left(\mathbf{r}-\mathbf{r}^{\kappa}(t)\right)\right\rangle \tag{13}$$

Replacing $M\dot{\mathbf{v}}$ with the instantaneous net force, $\mathbf{F}^{\kappa}(t)$, on molecule κ, moving the derivative operator ∇ outside the sum in the second term on the right, and noting that $\left\langle\sum_{\kappa=1}^{\mathscr{N}} M\mathbf{v}^{\kappa}\mathbf{v}^{\kappa}\delta\left(\mathbf{r}-\mathbf{r}^{\kappa}(t)\right)\right\rangle = \rho\mathbf{v}\mathbf{v}$, (13) becomes

$$(\rho \mathbf{v})_t + \nabla\cdot(\rho\mathbf{v}\mathbf{v}) = \left\langle \sum_{\kappa=1}^{\mathscr{N}} \mathbf{F}^{\kappa}(t)\,\delta\left(\mathbf{r}-\mathbf{r}^{\kappa}(t)\right)\right\rangle \tag{14}$$

Next, we apply Cauchy's[2] continuum mechanics-based argument to derive a second, equivalent ensemble average, SMS momentum equation. Thus, consider a SMS fluid system acted on by a combination of surface forces and volumetric body forces:

$$\frac{d}{dt}\int_{V_o(t)} \rho\mathbf{v}\,dV = \mathbf{F_{surf}}(t) + \mathbf{F_{vol}}(t) \tag{15}$$

where $V_o(t)$ is the time-varying volume of the single-molecule system, and where

$$\mathbf{F_{surf}}(t) = \oint_{A_o(t)} \mathbf{t}^{(\hat{\mathbf{n}})}\,dA \tag{16}$$

and

$$\mathbf{F_{vol}}(t) = \int_{V_o} \mathbf{f}(t)\,dV \tag{17}$$

Here, $A_o(t)$, is the area enclosing $V_o(t)$, $\mathbf{t}^{(\hat{\mathbf{n}})} = \mathbf{t}^{(\hat{\mathbf{n}})}(\mathbf{x},t)$, is the position- and time-dependent stress vector (field), acting at all points, $\mathbf{x}$, on $A_o(t)$, and $\mathbf{f} = \mathbf{f}(\mathbf{x},t)$, is the body force (field), likewise acting at all points within $V_o(t)$.

By Leibnitz's theorem, the first term in (15) can be written as

$$\frac{d}{dt}\int_{V_o(t)} \rho\mathbf{v}\,dV = \int_{V_o(t)} (\rho\mathbf{v})_{,t}\,dV + \int_{V_o(t)} \nabla\cdot(\rho\mathbf{v}\mathbf{v})\,dV \tag{18}$$

where, on the right side, the partial derivative with respect to time on the right is denoted with subscript t, and where, by the divergence theorem, the area integral, $\oint_{A_o(t)} \rho\mathbf{v}\mathbf{v}\cdot\hat{\mathbf{n}}\,dA$, is re-expressed as the second volume integral. Here, $\hat{\mathbf{n}}$ is the local outward unit normal vector on $A_o(t)$.

The continuum mechanical relationship, $t_i^{(\hat{n})} = \sigma_{ij}n_j$, connecting the stress vector to the stress tensor, σ_{ij}, is derived by application of momentum conservation to continuous, infinitesimal masses[2,10]. Here, the relationship holds for volumes that are infinitesimal on the single-molecule-scale. Thus, again via the divergence theorem, the instantaneous surface force for single-molecule-scale systems becomes:

$$\mathbf{F_{surf}}(t) = \int_{V_o(t)} \nabla\cdot\sigma\,dV \tag{19}$$

Collecting the volume integrals on the left side of (15) and applying the mean value theorem then leads to

$$\left[(\rho\mathbf{v})_t + \nabla\cdot(\rho\mathbf{v}\mathbf{v}) - \nabla\cdot\sigma - \mathbf{f}\right]V_o(t) = \mathbf{0} \qquad \text{as} \quad V_o(t)\to 0 \tag{20}$$

Since $V_o \neq 0$, the term in brackets, must equal zero, yielding a second version of the generic, ensemble average, single-molecule-scale momentum equation:

$$(\rho\mathbf{v})_t + \nabla\cdot(\rho\mathbf{v}\mathbf{v}) = \nabla\cdot\sigma + \mathbf{f} \tag{21}$$

Considering (21), we first note that this equation applies to *any* single-molecule-scale system, i.e, solid-, liquid-, gas-, or plasma-state, involving *any* molecular species. In order to apply the equation to a given molecular system, a single-molecule-scale constitutive relationship must be postulated. The analysis in the article, as well as that in Supplements 2 and 3, suggests that Newtonian viscosity and viscous shear stresses emerge on single-molecule length scales, and on the intermediate time-scale, τ_I. In addition,[1] shows that the Einstein-Stokes relationship, which requires that individual molecules are subject to a single-molecule-scale continuum viscous drag force identical to that on macroscopic spheres, well-predicts experimentally measured self-diffusion coefficients in liquid Ar, Kr, and Xe. Thus, we assume that the simplest constitutive relationship possible, applicable to Newtonian fluids, holds:

$$\sigma_{ij} = -P\delta_{ij} + \mu\left(v_{i,j} + v_{j,i}\right) + \mu_B \nabla \cdot \mathbf{v}\delta_{ij} \tag{22}$$

Here, P is the average of the three instantaneous single-molecule-scale principal stresses, μ is the dynamic viscosity, u_B is the bulk or dilatational viscosity, and δ_{ij} is the Kroenecker delta function[2,7]. Under single-molecule-scale equilibrium, as well as under weakly nonequilibrium conditions, (22) holds, and P corresponds to the single-molecule-scale equilibrium thermodynamic pressure. Note, while stress production by volumetric dilatation is minimal in continuum-scale liquids, due to inter-molecular voids, it likely plays a prominent role in SMS liquid systems.

On field-based and discrete descriptions of the intermolecular forces

Comparing (21) with (14), we arrive at an important equality:

$$\left\langle \sum_{\kappa=1}^{\mathscr{N}} \mathbf{F}^{\kappa}(t)\,\delta\left(\mathbf{r}-\mathbf{r}^{\kappa}(t)\right) \right\rangle = \nabla \cdot \sigma + \mathbf{f} \tag{23}$$

The significance of this relationship derives from the following features:

a) Any physically reasonable, discrete particle model of single-molecule-scale intermolecular forcing can be used to derive its field-based, ensemble average embodiment, as represented by the term on the left in (23). Examples of discrete particle force models include combined intra- and inter-molecular coulomb force models involving a target molecule system at $\mathbf{x}$, and molecules near $\mathbf{x}$[11], and pair-wise forces derived from the Lennard-Jones potential.

b) The process of recasting discrete particle models into ensemble average, field-based form, i.e., deriving a working version of $\left\langle \sum_{\kappa=1}^{\mathscr{N}} \mathbf{F}^{\kappa}(t)\,\delta\left(\mathbf{r}-\mathbf{r}^{\kappa}(t)\right) \right\rangle$, suitable for determining the single-molecule-scale velocity field, $\mathbf{v}(\mathbf{r},t)$, represents an open research problem[12]. We touch on this more in c), but note that the Green-Kubo machinery for determining the shear viscosity in terms of intermolecular forces[7–9,13] provides useful guidance.

c) The difficulty associated with deriving $\left\langle \sum_{\kappa=1}^{\mathscr{N}} \mathbf{F}^{\kappa}(t)\,\delta\left(\mathbf{r}-\mathbf{r}^{\kappa}(t)\right) \right\rangle$ from first principles arises from the central importance of quantum mechanical effects, including, for example, induced polarization in weak pair-wise interactions between non-polar molecules[11,14], and exchange interactions, arising from the exclusion principle[11], and which are expected to become important under high pressure conditions.

d) Crucially - and in light of the results in the article - indicating that single-molecule-scale viscous stresses and equilibrium pressures emerge on the sub-collision time-scale - a more accessible approach to closing the problem of finding the single-molecule-scale velocity field, $\mathbf{v}(\mathbf{r},t)$, centers on solution of (21).

e) In slight contrast with existing statements of molecular hydrodynamic momentum conservation, the present derivation clarifies the origin of the molecular-scale stress tensor, emphasizing the generic, field-based argument proposed by Cauchy[2].

Constitutive relations

For completeness, we restate the Newtonian, single-molecule-scale constitutive relationships assumed above. These are identical in form to N-particle system constitutive relationships[7]; we have simply adapted these to single-molecule-scale systems. Thus, we repeat (5) and (7):

$$m\langle n(\mathbf{r},t)\rangle = \rho(\mathbf{r},t) \tag{5}$$

$$\langle \mathbf{J}(\mathbf{r},t)\rangle = \rho(\mathbf{r},t)\,\mathbf{u}(\mathbf{r},t) \tag{7}$$

and add (22):

$$\sigma_{ij}(\mathbf{r},t) = -P\delta_{ij} + \mu\left[v_{i,j} + v_{j,i} - \frac{2}{3}\delta_{ij}v_{i,i}\right] \tag{22}$$

where detailed molecular-scale descriptions of the momentum current density, $\sigma_{ij}(\mathbf{r},t)$, is given, e.g, in[7–9,15].

Supplement 2: Single-molecule-scale stress fields and nonequilibrium statistical mechanics: Green-Kubo estimate of liquid viscosity

The arguments presented in Supplement 1, leading to single-molecule-scale, field-based conservation laws, stated in terms of ensemble average molecular-scale mass and momentum densities and currents, follow the same recipe used to derive continuum-scale conservation laws, and are thus, rigorously correct. However, in order to arrive at a mathematically-tractable, field-based model of single-molecule-scale dynamics, the six independent, single-molecule-scale, ensemble average stress tensor components must be restated in terms of the ensemble average SMS velocity field. As detailed in Supplement 1, we use the same assumption used to arrive at the continuum-scale Navier-Stokes equations by introducing a single-molecule-scale version of the continuum-scale Newtonian constitutive relationship, equation (22). Under SMS equilibrium, as well as under the weakly nonequilibrium conditions where (22) applies, P corresponds to the SMS equilibrium thermodynamic pressure. As detailed in[7], and independent of the demonstrated validity of (22) in continuum-scale flow problems, symmetry and physical arguments can be made that lead to (22), on any scale.

As a further test of the validity and consistency of a field-based, Navier-Stokes model of single-molecule-scale dynamics, this section uses a simple scaling argument to estimate, via a Green-Kubo relation[7,8,13], the single-molecule-scale dynamic viscosity in a generic nonpolar liquid. Since derivation of the Green-Kubo relations assumes existence of the Navier-Stokes equations, this example provides further insight into the physical nature of ensemble average, single-molecule-scale stress and velocity fields.

The Green-Kubo viscosity relation[8,9,13],

$$\mu = V\beta\int_0^\infty \langle P_{xy}(t)P_{xy}(0)\rangle dt \tag{24}$$

connects the shear viscosity, μ, to the ensemble-average, molecular-scale stress tensor, σ; see, e.g.[8,9,13] for details. As applied to continuum-scale Newtonian fluid flows, V is a volume large relative to the characteristic volume of individual molecules, $O\left(\sigma_o^3\right)$, and small relative to the characteristic dimensions of the flow, while the time-scale over which the correlation function, $\left\langle P_{xy}(t)P_{xy}(0)\right\rangle$, decays is on the order of the intermolecular collision time-scale, $\tau_c = \sigma_o/a$.

Define an SMS coordinate system with an origin at a given target molecule, initially in equilibrium. Due to a weak perturbation, a weak nonequilibrium momentum current appears in the vicinity of the molecule; consistent with convention, we label the direction of this current as the longitudinal direction, and define an x-axis in this direction. In addition, let the y-axis lie in a direction transverse to the x-axis. Over the ensemble of single molecules lying nearest the fixed origin, $\mathbf{r} = \mathbf{0}$, we assume that the *ensemble average* perturbation, at least on a single-molecule-scale, appears as a planar momentum current.

Next, use the SMS Newtonian constitutive relationship given in (22), $\langle P_{xy}\rangle = \sigma_{xy} = \mu u_{x,y}$, to express the transverse stress tensor correlation as

$$\left\langle P_{xy}(t)P_{xy}(0)\right\rangle = \mu^2\left\langle \frac{[u_1(\delta y,t) - u_1(0,t)]}{\delta y}\frac{[u_1(\delta y,0) - u_1(0,0)]}{\delta y}\right\rangle \tag{25}$$

Here, $u_1(\delta y,t)$ is the $x-$component of an individual molecule's velocity, evaluated at $y = \delta y$ and time, t, as observed in a given system within the ensemble. In addition, the evolution of u_1 is governed by Langevin's equation; see article. For clarity, the fixed $x-$ and $z-$coordinates of the two evaluation points used in the two partial derivatives are suppressed.

In order to evaluate these single-molecule-scale partial derivatives, we consider three physical features. First, we assume, again, that SMS viscosity emerges, in a sustained manner, on intermediate and longer time-scales, *in response to the appearance of persistent SMS transverse momentum currents,* i.e., sustained transverse SMS shear stresses. Second, and again based on the apparent existence of single-molecule-scale Stokes flow[1], the distance δy must on the order of the characteristic molecular diameter, $\delta y \approx \sigma_o$. Third, since significant dispersion-induced displacements of individual nuclei only occur on intermediate and longer time-scales, and assuming memory-free Langevin dynamics, the partial derivatives in (25), evaluated at $t = t$ and $t = 0$, are assumed statistically independent.

Thus, the sustained SMS transverse momentum current appears first, at $t = 0^+$, at $y = \delta y$. Prior to appearance of the momentum current, all molecules in the vicinity of $y = 0$ and of $y = \delta y$ are in local, thermal equilibrium, characterized by

coexistent high frequency, collective phonon modes, slow, single molecule diffusion, and resultant counter-flowing, isotropic momentum currents, that over the ensemble, exactly balance. In response to appearance of the sustained momentum current, however, molecules in the vicinity of $y = \delta y$ begin, at $t = 0^+$, to undergo Langevin motion, while those in the vicinity of $y = 0$, remain in equilibrium. Based on two features, we assume that single-molecule-scale motion is only correlated on length scales on the order of σ_o : i) Fast, dispersion-time-scale intermolecular dispersive forces and the preponderance of intermediate-time-scale two-body dispersive interactions randomize τ_I-scale dynamics of individual molecules. ii) Focusing on weak nonequilibrium perturbations, e.g., those produced by continuum-scale stress fields, sustained transverse momentum currents are weak enough to remain uncoupled from the background high frequency phonon field.

Thus, we can write the average in (25) as

$$\left\langle \frac{\left[u_1(\delta y,t)-u_1(0,t)\right]}{\delta y}\frac{\left[u_1(\delta y,0)-u_1(0,0)\right]}{\delta y}\right\rangle = \left\langle u_1(\delta y,t)\,u_1\left(\delta y,0^+\right)\right\rangle - \left\langle u_1(\delta y,t)\,u_1\left(0,0^+\right)\right\rangle - \\ - \left\langle u_1(0,t)\,u_1\left(\delta y,0^+\right)\right\rangle + \left\langle u_1(0,t)\,u_1\left(0,0^+\right)\right\rangle \tag{26}$$

The second and third ensemble averages above are zero since the intermediate time-scale, memory-free Langevin motion of molecules at $y = \delta y$ and $y = 0$, evaluated at the time instants shown, t and $t = 0^+$, are uncorrelated. Considering the fourth average, we choose δy to be on the order of a few molecular diameters, $= c_1\sigma_o$, where c_1 is an order 1 constant. Specifically, on time scales on the order of $\tau_I = 10^{-15}$ s, and in noble liquids, the ratio of the characteristic viscous penetration depth to the molecular diameter, $\sqrt{\nu\tau_I}/\sigma_o, \approx 0.1$ to 0.3. Thus, for c_o on the order of, say 2 or 3, molecules near $y = 0$, in an average sense, are not influenced by the transverse momentum current that appears at $y = \delta y$; thus, the fourth average in (25) is approximately zero.

Collecting results, and using the solution for a single realization of the molecule's random motion, as governed by the memory-free Langevin equation,

$$u_0 e^{\gamma t} + \int_0^t e^{-\gamma\left(t-t'\right)}\frac{F_1}{m}\left(t'\right)dt' \tag{27}$$

we obtain:

$$\int_0^\infty \left\langle P_{xy}(0)\,P_{xy}(t)\right\rangle dt \approx \frac{\mu^2}{\delta y^2}\left\langle u_1^2(\delta y,0)\right\rangle \int_0^\infty e^{-\gamma t'}dt' = \frac{\mu^2}{\delta y^2}\frac{u_0^2}{\gamma} = \frac{\mu}{3\pi\sigma_o^3 c_o^2\beta} \tag{28}$$

where $u_0 = u_1(\delta y,0)$, $\left\langle u_1^2(\delta y,0)\right\rangle = \beta = k_BT/m$, and $\gamma = 3\pi\mu\sigma_o$, and where we assume that the dispersive propulsive force, $F_1(t)$, and initial molecular velocity, $u_1(\delta y,0)$, are uncorrelated:

$$\int_0^t e^{-\gamma\left(t-t'\right)}\left\langle u_1(\delta y,0)\frac{F_1\left(t'\right)}{m}\right\rangle dt' = 0 \tag{29}$$

Comparison of the approximate result in (28) with the exact result[8,9,13]:

$$\int_0^\infty \left\langle P_{xy}(0)\,P_{xy}(t)\right\rangle dt = \frac{\mu}{V\beta} \tag{30}$$

leads to two important conclusions:

a) the proposed picture of single molecule, intermediate time-scale Langevin dynamics leads to a physically consistent viscosity prediction, and

b) consistent with our central argument that viscosity and viscous shear appears on single molecule scales, the characteristic dimension of the SMS system volume, $V^{1/3}$, is on the order of a few molecular diameters,

$$V^{1/3} = c_o^{2/3}(3\pi)^3\sigma_o \tag{31}$$

Supplement 3: Intermediate time-scale, single-molecule length scale emergence of viscosity

Three pieces of evidence suggest that, in noble liquids, viscosity emerges on time-scales, $\tau_{viscemergence}$, on the order of $\tau_I = O\left(10^{-15}\text{ s}\right)$:

a) The most direct evidence follows from the semi-empirical expression derived in the article for dynamic viscosity,

$$\mu = \frac{\sqrt{M\varepsilon}}{\sigma_o} \tag{32}$$

where ε, the characteristic dispersion energy between pairs of weakly interacting atoms, is shown to be frequency- and temperature-dependent:

$$\varepsilon = \frac{3}{4}h\nu_o\frac{\alpha^2}{r_{ab}^2} \tag{33}$$

and where $\alpha = \alpha(T)$ is a temperature-dependent polarizability and r_{ab} is the separation between the pair's nuclei.

Derivation of (33) invokes the Drude oscillator model[11], which replaces an n-electron, n-proton atom with a three-dimensional oscillator, comprised of a single negatively and a single positively charged particle. A quantum normal mode analysis of two weakly interacting Drude atoms[11,14] then leads to (33).

Crucially, (32) and (33) well-predict experimentally observed, temperature-dependent viscosities, for liquid Ar, Kr, Xe, N_2, and O_2, over each specie's range of liquid-state temperatures; see the article. Thus, estimating the fundamental ground state frequency, ν_o, as $\nu_o \approx E_I/h$[11] (where E_I is the ionization energy), recognizing that ν_o^{-1} sets the time-scale, τ_d, for dispersion-induced deformations of atomic electron distributions, and noting that (32) and (33) well-predict experimentally observed viscosities (see the article), we surmise that viscosity emerges on time-scales on the order of $10\ \tau_d$ to $10^2\ \tau_d\,(= \tau_I)$.

b) A loose upper bound on $\tau_{viscemergence}$ is provided by photon scattering and molecular dynamics simulations of the collective, long-time, i.e., hydrodynamic response of atomic liquids to (weak) external forcing[7,8]. Since, in noble liquids, well-defined viscously damped acoustic modes, as well as purely diffusive, viscously dissipated entropy modes are observed on time-scales, τ_c, on the order of the inverse collision frequency, $\nu_c^{-1} = \tau_c = \sigma_o/a = O\left(10^{-13}\text{ s}\right)$, then $\tau_{viscemergence} << \tau_c$.

c) A tighter upper bound on $\tau_{viscemergence}$ is obtained by consideration of recent work on phonon modes in liquids[16,17], where the approximate limiting (Frenkel) time-scale, τ_F, separating predominantly solid-like nonequilibrium response and liquid-like nonequilibrium response, is given by

$$\tau_F = \frac{\mu}{G_\infty} \tag{34}$$

and where G_∞ is the infinite frequency shear modulus. Importantly, since $\tau_F = O\left(10^{-14}\text{ s}\right)$, for most noble liquids[16], (34) implies that $\tau_{viscemergence} << \tau_F$.

Supplement 4: Simple liquid kinematic viscosities at fixed temperatures as determined by dispersion forces

As an initial test of the idea that viscosity in simple liquids can be estimated using the dispersion energy, ε_d, in

$$\mu \approx \frac{\sqrt{\varepsilon M}}{\sigma^2} \tag{35}$$

we use a more rigorous expression for ε_d,

$$\varepsilon_d = \frac{C}{r_{ab}^6} \tag{36}$$

obtained by a quantum mechanical second order perturbation calculation of the interaction energy between pairs of spherical molecules[11,14], where London called C the attractive constant. Using (36) in (35), approximating r_{ab} as the molecular diameter,

Comparison of experimental and theoretical kinematic viscosities				
Specie	C_H/C_L $\mathrm{erg \cdot cm^6 X10^{60}}$	σ 10^{-10} m	ν_H/ν_L $\mathrm{m^2\ s^{-1} X10^8}$	ν_{exp} [ref] $\mathrm{m^2 s^{-1} X10^8}$
Ne	5.64/4.69	2.86	5.02/4.58	5.79[18]
Ar	66.7/55.6	3.47	8.35/7.62	9.9[18]
Kr	130.0/110.6	3.61	7.42/6.84	5.7[19]
Xe	280/234	4.06	6.89/6.3	5.5[19]
H_2	15.5/13.3	2.93	25.2/23.3	14.7[18]
N_2	66.8/61.8	3.75	8.5/8.2	14.[19]
O_2	48.8/43.6	3.43	8.1/7.7	13.[19]

Table 1. Due to the spread in published magnitudes of the attractive constant, C, the magnitudes reported by Hirschfelder[11], C_H, and London[14], C_L, are included. Corresponding approximate kinematic viscosities, obtained using (37), are listed as ν_H and ν_L. The experimental conditions and techniques used to measure ν_{exp} are described in the cited references.

σ, and dividing by $M/\left(\pi\sigma^3/6\right)$ we obtain an order of magnitude estimate of the kinematic viscosity, stated in terms of molecular parameters:

$$\nu \approx \sqrt{\frac{C}{\sigma^6 M}}\sigma \tag{37}$$

A comparison of experimentally measured and estimated magnitudes of ν obtained by (37) are shown in Table 1. Importantly, while experimental measurements of ν are subject to significant uncertainty, this initial rough comparison, combined with the approximate model of dispersion outlined in the paper, suggests that observed temperature variations in simple liquid viscosities can be explained in terms of temperature-dependent variations in polarizability. Note that temperature-dependent variations in molecular spacing, σ, estimated using thermal expansion coefficients, are too large to explain observed viscosity variations. While the average molecular spacing increases with temperature, this has the effect of reducing the average dispersive interaction time between molecular pairs, but doesn't appreciably change the magnitude of the dispersive force.

Supplement 5: Illustration - using sum rules to develop short time scale collective dynamics models

As proposed in Table 2, for time scales longer than the Frenkel scale, $t \gtrsim \tau_F$, where the solid-like properties extant on $\tau_d \lesssim t \lesssim \tau_F$, begin to give way to 'slushy', combined solid- and fluid-like dynamics over $\tau_F \lesssim t \lesssim \tau_c$, the friction force on individual molecules can be reasonably assumed to either follow the modified Stokes drag law, or during scattering experiments, a memory-dependent drag law. However, in this slushy regime, specification of the random force remains problematic. In this Supplement, we carry out a preliminary investigation of *collective,* ensemble average dynamics extant on this time scale. Our objective centers on gaining insight into the effect of phonon dynamics, dominant on $\tau_d \lesssim t \lesssim \tau_F$, on slushy collective dynamics over $\tau_F \lesssim t \lesssim \tau_c$. In addition, this example illustrates the use of sum rules[7,8,20] for developing and testing hydrodynamic response models.

The model we propose combines ensemble average conservation statements for microscale mass and momentum density, $\langle n(\mathbf{r},t)\rangle$ and $\langle \mathbf{j}(\mathbf{r},t)\rangle$:

$$\frac{\partial \langle n(\mathbf{r},t)\rangle}{\partial t} + \nabla \cdot \langle \mathbf{j}(\mathbf{r},t)\rangle \tag{38}$$

$$\frac{\partial \langle \mathbf{j}(\mathbf{r},t)\rangle}{\partial t} = -\frac{1}{M}\nabla P(\mathbf{r},t) + \nu \nabla^2 \langle \mathbf{j}(\mathbf{r},t)\rangle \tag{39}$$

where

$$n(\mathbf{r},t) = \sum_{i=1}^{N} \delta\left(\mathbf{r} - \mathbf{r_i}(t)\right) \tag{40}$$

and

$$\mathbf{j}\left(\mathbf{r},t\right)=\sum_{i=1}^{N}\mathbf{v_i}\delta\left(\mathbf{r}-\mathbf{r_i}\left(t\right)\right) \tag{41}$$

and where $\langle\cdot\rangle$ denotes an ensemble average. See, e.g.,[8, 20].

In order to explicitly couple averaged, collective dynamics to intermolecular forces and single molecule-scale structure, we express the pressure in a form appropriate to (isotropic) interacting systems[21]:

$$P\left(\mathbf{r},t\right)=\langle n\left(\mathbf{r},t\right)\rangle k_B T\left(\mathbf{r},t\right)- \\ -\frac{\langle n\left(\mathbf{r},t\right)\rangle^2}{6}\int_0^\infty 4\pi\tilde{r}^3\frac{\partial u\left(\tilde{r};\langle n\left(\tilde{r},t\right)\rangle\right)}{\partial\tilde{r}}g\left(\tilde{r}\right)d\tilde{r} \tag{42}$$

where the strong dependence of the intermolecular potential, u, on the (local) average density, $\langle n\left(\mathbf{r},t\right)\rangle$, is emphasized, and where g is the pair distribution function[7, 8, 20].

The physical conditions and assumptions in this model are as follows: a) no scattering particles are present, and on the continuum scale, the liquid either flows or remains static; b) we focus on the collective, ensemble average response of a system of molecules to either a spontaneous (thermal) or externally imposed perturbation; c) focusing on dynamics extant on both on the slushy time scale, as well as the near-collision time scale, $t=\mathrm{O}\left(\tau_c\right)$, the fluid obeys the Newtonian constitutive assumptions[7]; and d) the dynamic viscosity, $\mu=\nu\rho_o$ is fixed, where ν and ρ_o are, respectively, the kinematic viscosity and the equilibrium mass density. The main caveat: The model assumes that slushy regime dynamics can be reasonably modeled using a fluid regime model. In reality, given the dominance of phonon dynamics over $\tau_d\lesssim t\lesssim\tau_F$, we expect that a more realistic model will incorporate viscoelastic constitutive relationships; this question to be pursued in future work. By contrast, on near-collision and longer time scales, the model is appropriate[8].

Sum rules provide a powerful tool for developing and testing models of collective dynamics[7, 8, 13, 20]. In particular, the short-time scale model response, as captured by the intermediate scattering function, $F\left(k,t\right)$, must exhibit short-time behavior given by[8]:

$$F\left(k,t\right)=\omega^o\left(k\right)-\frac{t^2}{2!}\omega^2\left(k\right)+\frac{t^4}{4!}\omega^4\left(k\right)-\ldots \tag{43}$$

where

$$\omega^n\left(k\right)=\frac{1}{2\pi}\int_{-\infty}^{\infty}\tilde{\omega}^n S\left(k,\tilde{\omega}\right)d\tilde{\omega} \tag{44}$$

and where

$$S\left(k,\omega\right)=\int_{-\infty}^{\infty}\langle n_k^*\left(0\right)n_k\left(t\right)\rangle\exp\left(-i\omega t\right)dt \tag{45}$$

is the dynamic structure factor and $n_k\left(t\right)$ is the Fourier transform of the number density,

$$n_k\left(t\right)=\int_{-\infty}^{\infty}n\left(\mathbf{r},t\right)\exp\left(i\mathbf{k}\cdot\mathbf{r}\right)d\mathbf{r} \tag{46}$$

(43), corresponding to the short-time Taylor expansion of $F\left(k,t\right)$, imposes a rigorous set of structural and dynamical constraints, via the frequency moments in (44), on the short-time response predicted by any potential hydrodynamic model. Thus, given a theoretical solution for $n_k\left(t\right)$, a corresponding structure factor, $S\left(k,\omega\right)$, can be calculated, and the frequency moments in (44) determined. The first two predicted moments must satisfy the following relationships[7, 8]:

$$\omega^0\left(k\right)=S\left(k\right)=\frac{1}{2\pi}\int_{-\infty}^{\infty}S\left(k,\tilde{\omega}\right)d\tilde{\omega} \tag{47}$$

$$\omega^2\left(k\right)=k^2 v_o^2 \tag{48}$$

where $v_o^2=k_b T/M$ is the thermal speed.

Following algebra outlined in[8], use (39) to eliminate $\langle \mathbf{j}(\mathbf{r},t)$ from (38):

$$\frac{\partial^2 \langle n(\mathbf{r},t)\rangle}{\partial t^2} - \nu\nabla^2 \frac{\partial \langle n(\mathbf{r},t)\rangle}{\partial t} = \frac{1}{M}\frac{\partial P}{\partial \langle n\rangle}_o \nabla^2 \langle n(\mathbf{r},t)\rangle \tag{49}$$

where the subscript 'o' on the right side denotes the local equilibrium value of the partial derivative. Using (42) in (49) and taking the spatial Fourier transform then yields:

$$\frac{\partial^2 \langle n(k,t)\rangle}{\partial t^2} + \nu k^2 \frac{\partial \langle n(k,t)\rangle}{\partial t} + k^2\left[v_o^2 - c_u^2\right] = 0 \tag{50}$$

where

$$c_u^2 = \frac{1}{M}\frac{\partial}{\partial \langle n\rangle}\left[\frac{\langle n(\mathbf{r},t)\rangle^2}{6}\int_0^\infty 4\pi\tilde{r}^3 \frac{\partial u(\tilde{r};\langle n(\tilde{r},t)\rangle)}{\partial \tilde{r}} g(\tilde{r})\,d\tilde{r}\right] \tag{51}$$

Solving (50) for $\langle n(k,t)\rangle$, then checking the solution against the first frequency moment in (47) shows that the solution obeys this constraint for all k. Next, using the solution in (48) leads to:

$$S(k)k^2\left(c_u^2 - v_o^2\right) = k^2 v_o^2 \tag{52}$$

Focusing on collective dynamics where $k \to 0$, and noting the limit[8] $S(k\to 0) = v_o^2/a_T^2$, where a_T is the isothermal sound speed, we obtain:

$$c_u^2 - v_o^2 = a_T^2 \quad k \to 0 \tag{53}$$

Finally, since $S(k)$ approaches 0 as $k \to 0$[8], then $v_o^2 << a_T^2$, and we find that $c_u \approx a_T$. Physically, assuming fluid-like behavior dominates viscoselastic dynamics within the slushy regime, this shows that multi-molecule interactions, captured by the second term in (42), become organized on long length scales, propagating multi-molecule scale fluctuations and disturbances via sound waves. In contrast to the viscously damped acoustic modes that emerge on time scales long relative to τ_c[7,8,20,22], i.e., low frequency acoustic modes, on the short time scale associated with slushy dynamics, viscous dissipation plays no role in sound wave propagation.

While this result is physically interesting, the important point is that a variety of hydrodynamic models, incorporating, for example, viscoelastic behavior on the slushy time scale, can be introduced for studying (ensemble average) dynamics on any of the three time scales characterizing the sub-collision scale. As another example, when one approaches and crosses into the solid-like regime, $\tau_d \lesssim t \lesssim \tau_F$, elastic constitutive relations can be assumed and the intermolecular potential, $u(r,n)$, can be cast in terms of the phonon internal energy[16]. Importantly, this line of attack may lead to much improved understanding of collective dynamics in nonpolar liquids, on these short time scales.

References

1. Edward, J. T. Molecular volumes and the stokes-einstein equation. *J. Chem. Educ.* **47**, 261–270, DOI: https://doi.org/10.1021/ed047p261 (1970).
2. Panton, R. L. *Incompressible Flow* (Wiley, 2013).
3. Keanini, R. G. *et al.* Macroscopic liquid-state molecular hydrodynamics. *Sci. Reports* **7**, 1–10, DOI: https://doi.org/10.1038/srep41658 (2017).
4. Fleischhauer, E., Dahlberg, J. L., Solomon, J. M., Keanini, R. G. & Tkacik, P. T. Kinematic viscosity measurement of granular flows via low reynolds number cylinder drag experiment. *Meas. Sci. Technol.* **30**, 055904, DOI: https://doi.org/10.1088%2F1361-6501%2Fab08cf (2019).
5. Mullany, B. *et al.* The application of computational fluid dynamics to vibratory finishing processes. *CIRP Annals* **66**, 309–312, DOI: https://doi.org/10.1016/j.cirp.2017.04.087 (2017).
6. Cini-Castagnoli, G. & Ricci, F. P. Self-diffusion in liquid argon. *J. Chem. Phys.* **32**, 19–20, DOI: https://doi.org/10.1063/1.1700899 (1960).
7. Forster, D. *Hydrodynamic Fluctuations, Broken Symmetry, and Correlation Functions* (Perseus, 1990).
8. Boon, J. P. & Yip, S. *Molecular Hydrodynamics* (McGraw Hill, 1980).
9. Evans, D. J. & Morriss, G. P. *Statistical Mechanics of Nonequilibrium Liquids* (Cambridge University Press, 2008).

10. Mase, G. T., Smelser, R. E. & Rossmann, J. S. *Continuum Mechanics, 4th ed.* (Taylor Francis, 2020).

11. Hirschfelder, J. O., Curtiss, C. F. & Bird, B. R. *Molecular Theory of Gases and Liquids* (Wiley, 1954).

12. Lewars, E. G. *Computational Chemistry, 3rd ed.* (Springer Nature, 2016).

13. Kubo, R., Toda, M. & Hashitsume, N. *Statistical Physics II: Nonequilibrium Statistical Mechanics* (Springer-Verlag, 1991).

14. London, F. The general theory of molecular forces. *Transactions Faraday Soc.* **33**, 8b–26, DOI: https://doi.org/10.1039/TF937330008B (1937).

15. Berne, B. J. & Pecora, R. *Dynamic Light Scattering* (Wiley, 1976).

16. Bolmatov, D., Brazhkin, V. V. & Trachenko, K. The phonon theory of liquid thermodynamics. *Sci. Reports* **2**, 421, DOI: https://doi.org/10.1038/srep00421 (2012).

17. Trachenko, K. & Brazhkin, V. V. Collective modes and thermodynamics of the liquid state. *Reports on Prog. Phys.* **79**, 016502, DOI: https://doi.org/10.1088/0034-4885/79/1/016502 (2015).

18. Agrawal, G. M. & Thodos, G. Kinematic viscosity of cryogenic fluids in their saturated liquid state. *Can. J. Chem. Eng.* **50**, 557–560, DOI: https://doi.org/10.1002/cjce.5450500423 (1972).

19. Haley, H. J. M., McCarty, R. D. & Haynes, W. M. The viscosity and thermal conductivity coefficients for dense gaseous and liquid argon, krypton, xenon, nitrogen and oxygen. *J. Phys. Chem. Ref. Data* **3**, 979–1018, DOI: https://doi.org/10.1063/1.3253152 (1974).

20. Hansen, J. P. & McDonald, I. R. *Theory of Simple Liquids with Applications to Soft Matter, 4th ed.* (Elsevier, 2013).

21. Pathria, R. K. & Beale, P. D. *Statistical Mechanics, 3rd ed.* (Academic Press, 2011).

22. Lovesey, S. W. & Springer, T. *Dynamics of solids and liquids by neutron scattering* (Springer-Verlag, 1977).
